# Phytochemical Profile and Biological Activities of *Baccharis dracunculifolia* DC—Aerial-Parts Extract: In Vitro Evaluation and Predictive Analyses

**DOI:** 10.3390/ph19081162

**Published:** 2026-07-25

**Authors:** Zilda Cristiani Gazim, Filipa Mandim, Josiana Vaz, Lillian Barros, Gabriel Augusto Rodrigues Beirão, Gabriel Ribeiro da Silva, Annye Vitória Moraes, Simone Francisca de Paula, Lidiane Nunes Barbosa, Beatriz Cervejeira Bolanho Barros, Daniela Dib Gonçalves, Juliana Silveira do Valle, Antonio Laverde Junior, Arquimedes Gasparotto Junior

**Affiliations:** 1 Graduate Program in Biotechnology Applied to Agriculture, Universidade Paranaense, Umuarama 87502-210, Brazil; gabriel.beirao@edu.unipar.br (G.A.R.B.); jsvalle@prof.unipar.br (J.S.d.V.); 2 Graduate Program in Animal Science with Emphasis on Bioactive Products, Universidade Paranaense, Umuarama 87502-210, Brazil; gabriel.200253@edu.unipar.br (G.R.d.S.); danieladib@prof.unipar.br (D.D.G.); 3 Graduate Program in Medicinal and Phytotherapeutic Plants in Primary Care, Universidade Paranaense, Umuarama 87502-210, Brazil; simone20277@edu.unipar.br (S.F.d.P.); lidianebarbosa@prof.unipar.br (L.N.B.); 4 CIMO, Laboratório Associado para a Sustentabilidade e Tecnologia em Regiões de Montanha (SusTEC), Instituto Politécnico de Bragança, Campus de Santa Apolónia, 5300-253 Bragança, Portugal; filipamandim@ipb.pt (F.M.); josiana@ipb.pt (J.V.);; 5 Laboratório Associado para a Sustentabilidade e Tecnologia em Regiões de Montanha (SusTEC), Instituto Politécnico de Bragança, Campus de Santa Apolónia, 5300-253 Bragança, Portugal; 6 Faculty of Health Sciences, Federal University of Grande Dourados, Dourados 79825-070, Brazil; anahvmoraes@gmail.com (A.V.M.); arquimedesjunior@ufgd.edu.br (A.G.J.); 7 Postgraduate Program in Sustainability, Universidade Estadual de Maringá, Campus Umuarama, Umuarama 87020-900, Brazil; bcbolanhobarros@uem.br; 8 Laboratório de Química Orgânica e Biomateriais, Departamento de Química, Universidade Tecnológica Federal do Paraná, Campus Londrina, Londrina 86036-700, Brazil; aljunior@utfpr.edu.br

**Keywords:** Alecrim-do-campo, phenolic compounds, PASS prediction, ADMET, antiproliferative activity, antioxidant activity, nitric oxide inhibition

## Abstract

**Background and Objectives:** *Baccharis dracunculifolia* DC. (Asteraceae), the main botanical source of Brazilian green propolis, is recognized for its high content of bioactive secondary metabolites. Given this potential, this study aimed to characterize the chemical profile of the crude extract (CE) from the aerial parts of *B. dracunculifolia* and to investigate its anti-inflammatory, antiproliferative, antioxidant, and photoprotective properties. Predictive computational analyses were used to assist the interpretation of the experimental findings. **Methods:** The CE was obtained by dynamic maceration with ethanol and chemically characterized by UHPLC-MS/MS using external calibration curves. Anti-inflammatory activity was evaluated by inhibiting nitric oxide (NO) in RAW 264.7 macrophages, while cellular antioxidant activity (CAA) was determined in the same model. Antiproliferative activity was evaluated against the human tumor cell lines AGS, Caco-2, MCF-7, and NCI-H460, as well as non-tumor VERO cells. Additionally, antioxidant potential was investigated using classical colorimetric methods (DPPH, FRAP, and ABTS). The extract was also quantified for total phenolic and flavonoid content, as well as sun protection factor (SPF). Furthermore, complementary computational analyses included PASS prediction, SwissTargetPrediction, Gene Ontology enrichment using PANTHER, SwissADME profiling, and toxicity prediction with ProTox-III. **Results:** UHPLC-MS/MS analysis revealed a profile rich in flavonoids and phenolic acids and led to the identification of 14 compounds not previously reported in *B. dracunculifolia* according to the literature examined, notably the flavonoid morin and the phenylpropanoid coniferaldehyde detected at comparatively high concentrations (>600 µg/g). CE inhibited NO production (IC_50_ = 62.00 µg/mL) suggesting anti-inflammatory activity, and reduced intracellular oxidation by 81% (at 2000 µg/mL) in RAW 264.7 macrophages. The extract showed total phenolic content (83.62 to 540.40 µg gallic acid equivalents/mg of CE) and high levels of flavonoids (472.00–515.00 µg quercetin equivalents/mg of CE), antioxidant activity in the DPPH assay (IC_50_ = 0.86 mg/mL), and relevant photoprotective potential (SPF = 7.79–19.30). Antiproliferative activity was moderate to weak (GI_50_ = 165.00–257.00 µg/mL). In silico analyses identified predicted activities, molecular targets, and enriched biological processes related to redox homeostasis, inflammation, apoptosis, and cellular responses to UV radiation, suggesting biological functions potentially associated with the identified metabolites. **Conclusions:** The crude extract of *B. dracunculifolia* demonstrated significant cellular anti-inflammatory and antioxidant activities, likely associated with its phenolic composition and the presence of metabolites reported in this study for the first time in this species, particularly morin and coniferaldehyde. These findings expand the phytochemical knowledge of *B. dracunculifolia* and reinforce its potential as a source of bioactive compounds for pharmaceutical, nutraceutical, and photoprotective applications.

## 1. Introduction

*Baccharis dracunculifolia* DC. (Asteraceae), popularly known as “Vassourinha” or “Alecrim-do-campo”, is a medicinal bush widely distributed in Argentina, Uruguay, Brazil, Paraguay, Bolivia, and Peru [1,2]. In Brazil, it represents the principal botanical source of “Brazilian green propolis” produced by *Apis mellifera*, one of the best-characterized propolis types worldwide, whose chemical composition, quality markers, and several biological activities have been extensively documented [3].

Extensive phytochemical and pharmacological investigations conducted over the last decades have established the chemical composition, quality markers, and diverse biological activities of *B. dracunculifolia* [4]. The aerial parts of *B. dracunculifolia* contain a wide range of secondary metabolites associated with antioxidant [5,6], anti-inflammatory [7,8], antimicrobial [9,10], antiviral [11], gastroprotective [12,13,14], and several other pharmacological activities.

Found naturally in thickets, roadsides, pastures, forests, and sandbanks, this species is considered invasive and is frequently eliminated because it competes with native vegetation. Despite this, due to its rapid vegetative growth, its use is recommended in the recovery of degraded areas [4,15]. In addition to its ecological importance, the plant is widely used in traditional medicine by various communities, being used in the treatment of malaria, parasitic infections, wounds, skin ulcers, fever, and gastrointestinal disorders. It is also used as an antispasmodic, diuretic, analgesic, and in the treatment of diabetes, as well as showing activity against bacterial and fungal infections [9,16].

The leaves and flowers produce an essential oil predominantly composed of terpenoids [17], whereas crude extracts from the aerial parts are characterized by high levels of phenolic acids, phenylpropanoids (including simple, prenylated, cinnamic, and chlorogenic acid derivatives), flavonoids, and diterpenes, particularly those with neo-clerodane, labdane, and kaurane skeletons [18,19,20,21].

Numerous phytochemical studies have shown that many of these metabolites are also present in Brazilian green propolis, reinforcing the close chemical relationship between the plant and its resinous product. Among the best-characterized constituents are artepillin C, baccharin, drupanin, caffeic acid derivatives, and several flavonoids, compounds widely recognized as major contributors to the antioxidant, anti-inflammatory, antimicrobial, and other pharmacological properties associated with both *B. dracunculifolia* and Brazilian green propolis [1,9,18,19,20,21].

Although the phytochemical composition and pharmacological properties of *B. dracunculifolia* have been extensively investigated, previous studies have generally investigated these aspects independently. Comprehensive studies integrating quantitative metabolite profiling, multiple biological assays, and computational target prediction using the same chemically characterized crude extract remain scarce. This separation makes it difficult to examine how the chemical composition of a given extract relates to its biological responses and to explore which metabolites may be associated with the observed activities.

Therefore, the present study combined quantitative phytochemical characterization of a *B. dracunculifolia* crude extract with multiple biological assays. Metabolites were identified and quantified by UHPLC-MS/MS using authentic standards, followed by experimental biological assays. PASS, SwissTargetPrediction, Gene Ontology enrichment, and ADMET analyses were subsequently used as complementary tools to explore biological activities, molecular targets, and biological processes potentially associated with the identified metabolites [22,23].

In addition to phytochemical characterization, this study included biological activities that have been little explored for this species, such as cellular antioxidant activity (CAA), inhibition of nitric oxide (NO) production, antiproliferative activity against tumor cell lines not previously investigated for *B. dracunculifolia* [24], and photoprotective potential based on SPF, UVA/UVB ratio, and critical wavelength (λc) [25].

By integrating phytochemical characterization, multiple in vitro biological assays, and complementary computational analyses within a unified experimental framework, this study provides an integrated assessment of the metabolite profile and biological activities of *B. dracunculifolia* crude extracts. The computational analysis was used to support the interpretation of experimental findings and to explore biological functions potentially associated with the identified metabolites. Together, these results complement the existing literature on this medicinal species while further supporting its close chemical relationship with Brazilian green propolis.

## 2. Results and Discussion

The biological activities attributed to *B. dracunculifolia* are largely related to the synergistic and additive effects of its phenolic compounds, which constitute the major fraction of both the crude extract obtained from the plant’s aerial parts and green propolis [26]. Given that *B. dracunculifolia* is the primary botanical source of green propolis; the aerial parts were collected at the onset of the period when bees begin to visit the plant and gather resin. This strategy aimed to obtain an extract with a phytochemical profile that more closely resembles the botanical source material available to bees during green propolis production, particularly for phenolic compounds. Thus, the first stage of this study involved the chemical characterization of the crude extract from *B. dracunculifolia* aerial parts using UHPLC-MS/MS, with an emphasis on the identification and annotation of phenolic compounds.

The retention times and spectral profiles of the extract components were compared with 38 authentic reference standards of various phenolic and polyphenolic compounds, as well as three organic acids and four common nitrogenated compounds, thereby allowing the identification and quantification of 26 secondary metabolites using calibration curves, as presented in Table 1. Several of these compounds have already been reported in other studies with extracts of *B*. *dracunculifolia* [3,10,21,26,27,28]. However, some of the identified compounds are being reported for the first time in this species, to the best of our knowledge: malic, quinic, *p*-hydroxybenzoic, α-resorcylic, and nicotinic acids; coumarin; *p*-hydroxybenzaldehyde; vanillin; isovanillin; coniferaldehyde; luteolin; (-)-epicatechin; morin; and caffeine. Although luteolin is being reported for the first time in *B*. *dracunculifolia*, this known flavone has already been identified in other *Baccharis* species (*B. bigelovii*, *B. incarum*, *B. illinita*, *B. microcephala*, *B. nitida*, *B. pteronioides*, *B. trimera*) [19,29,30].

According to our results, phenylpropanoids constituted the most abundant class present in the *B*. *dracunculifolia* extract, with approximately 3000 µg/g, with coniferaldehyde (>600 µg/g) and chlorogenic (>600 µg/g), *p*-coumaric (>600 µg/g), and caffeic (379.91 µg/g) acids as major components, in accordance with Bastos and Arruda [31], who state that phenolic compounds are represented by coumarins, phenolic acids, phenylpropanoids (simple and prenylated), and flavonoids (aglycone and glycosides). Búfalo et al. [32] also reported that the main components in the aerial parts of *B*. *dracunculifolia* were derived from cinnamic acids, especially ferulic, caffeic, and *p*-coumaric acids.

Flavonoids emerged as the second most abundant class in the extract, with a value greater than 1740 µg/g, highlighting the presence of the flavanone naringenin (569.51 µg/g), and the flavonols morin (>600 µg/g) and rutin (490.08 µg/g). According to Maróstica et al. [33], this species exhibits a great diversity of flavonoids. In particular, the identification of a high concentration of morin (>600 µg/g) in the present study was a positive surprise, since this aglycone is an isomer of quercetin (C_15_H_10_O_7_), one of the most common flavonols in higher plants, and had not yet been reported in the genus *Baccharis*. Quercetin and morin differ in the substitution pattern of ring B, where the hydroxyl groups occupy the 3′,4′ and 2′,4′ positions, respectively. The presence of morin at a concentration much higher (>600 µg/g) than quercetin (38.7 µg/g) led us to question whether other studies that did not use authentic reference standards may have been mistaken in identifying or quantifying quercetin, since these isomers exhibit similar MS/MS fragmentation patterns and close retention times. Our results also revealed the presence of three other pairs of isomers in this extract: salicylic and *p*-hydroxybenzoic acids, which differ from each other by *ortho* and *para* substitution, respectively; vanillin and isovanillin, which differ by the hydroxyl and methoxyl groups occupying positions 4,3 and 3,4 of the aromatic ring, respectively; and protocatechuic and α-resorcylic acids, where the hydroxyl groups occupy positions 3,4 and 3,5, respectively. Among the two natural forms of catechin, only the diastereoisomer (-)-epicatechin has been identified.

Although the number of identified and quantified compounds is large, some important phenolic compounds that are usually present in green propolis and the aerial parts of *B. dracunculifolia*, such as drupanin, baccharin, and artepillin C, were not investigated in this work due to difficulties in obtaining authentic samples of these compounds. Therefore, we cannot rule out the possibility of these compounds having influenced the evaluated activities. Even so, we consider that chemical identification by UHPLC-MS/MS proved adequate for the proposed objectives, based on the agreement between the retention times and the characteristic MS/MS transitions of the analytes and their respective reference standards, providing a high degree of confidence in the attribution of the compounds, specially some isomers. Furthermore, the quantitative analyses were based on external calibration curves prepared from standard mixtures at concentrations ranging from 10 to 250 µg/L. Linear regression analyses demonstrated satisfactory linearity across the entire working range, with correlation coefficients (R^2^) greater than 0.98 for all analytes.

The flavonoid content (472.00–515.00 µg quercetin equivalents/mg of extract) (Table 2) was consistent with the results obtained in the chemical characterization by UHPLC-MS/MS, which revealed a quantitative predominance of this class of metabolites in the *B. dracunculifolia* extract. Furthermore, the high abundance of phenylpropanoids identified (Table 1) may have contributed to the response observed in the total flavonoid assay (Table 2), reflecting the close biosynthetic relationship between these classes of compounds. Phenylpropanoids constitute fundamental intermediates of the phenylpropanoid pathway, originating 4-coumaroyl-CoA, a central precursor in flavonoid biosynthesis [34]. Thus, the observed chemical profile suggests an active phenolic metabolism, characterized by the concomitant accumulation of phenylpropanoids and flavonoids.

Thus, the colorimetric assays employed indicated a significant contribution of phenolic compounds, especially flavonoids, to the phytochemical profile of the extract. These results are in agreement with previous studies, which also reported high levels of these metabolites in *B. dracunculifolia* extracts [35,36].

Phenolic and polyphenolic compounds are key in neutralizing reactive oxygen species (ROS) and protecting cells from oxidative damage [37]. These compounds in plant extracts can interact synergistically, additively, or antagonistically [38]. Analytical protocols typically measure total antioxidant activity, reflecting the combined bioactivity of these compounds. However, since no single method captures all aspects of antioxidant capacity, we employed multiple assays to comprehensively assess the antioxidant potential of the *B. dracunculifolia* aerial extract. As emphasized by Alam et al. [39], the results of antioxidant assessments may vary depending on the method used, the chemical nature of the sample, and the interactions among compounds in the extract. Therefore, the combined use of DPPH^•^, ABTS^•+^, and FRAP assays increases the reliability and interpretative depth of the antioxidant profile [40].

We present in Table 3 the results regarding the regenerative action of DPPH^•^ and ABTS^•+^ free radicals, as well as the ferric reducing antioxidant power (FRAP) against the crude extract of *B*. *dracunculifolia*. Of the three assays evaluated, the DPPH^•^ assay showed the strongest antioxidant effect, regenerating 50% of the free radical at a concentration of 0.86 mg/mL. For the ABTS^•+^ and FRAP assays, the results were not significantly different from the trolox control.

These results reflect the antioxidant capacity of the extract components and the diversity of mechanisms involved in this process, suggesting that the set of secondary metabolites, especially phenolic and polyphenolic compounds, may act in different ways to confer antioxidant properties.

The production of free radicals, such as NO^•^ and O_2_^•−^, is a continuous, physiological process in which they participate in numerous metabolic processes, contributing to the body’s defense mechanisms; however, in excess, they can compromise cellular functions [41]. Excessive production of these free radicals can oxidize biomolecules, impair their biological functions, and disrupt homeostasis, resulting in oxidative and nitrosative damage to cells and tissues. The imbalance between these free radicals is considered an important factor in the aging process, as it is responsible for mitochondrial dysfunction, cellular apoptosis, and other pathological changes associated with aging, as well as triggering various pathological events such as inflammation, which in turn is also involved in cardiovascular, carcinogenic, and neurodegenerative processes [42]. In the inflammatory process, some cells are activated, among them macrophages, which produce inflammatory mediators, including nitric oxide, superoxide anion, transcription factors, and cytokines [43].

In this sense, our study reports, for the first time, the evaluation of cellular antioxidant activity and inhibition of nitric oxide production in RAW 264.7 murine macrophages in vitro, which are commonly used as indicators of antioxidant and anti-inflammatory potential in vitro. The crude extract of *B*. *dracunculifolia* suppressed the production of nitric oxide (NO^•^) and superoxide radical (O_2_^•−^) in macrophage culture, as shown in Table 4.

Regarding cellular antioxidant activity (CAA), the crude extract of *B*. *dracunculifolia* showed 81% inhibition at the maximum concentration tested, a result close to that of the quercetin control (93%). The high percentage of inhibition observed evidenced the presence of components in the extract capable of acting as antioxidant protective agents, maintaining biological efficacy within a complex cellular environment. The crude extract also inhibited nitric oxide (NO^•^) production, with an IC_50_ of 62.00 µg/mL, a value only 9.8 times lower than that of dexamethasone (positive control). Several authors have investigated the anti-inflammatory action of *B*. *dracunculifolia* extracts using different in vivo and in vitro models [1]. Cestari et al. [44] evaluated the anti-inflammatory potential of ethyl acetate extract from the aerial parts of *B*. *dracunculifolia* in an in vivo colitis model. According to the authors, this extract reduced colon damage and enzymatic activity of biochemical markers of inflammation after the first week in the chronic colitis model, leading the authors to suggest that the anti-inflammatory activity is related to phenylpropanoid compounds, such as caffeic acid, *p*-coumaric acid, drupanin, baccharin, and artepillin C, the main component of the extract. Some of these phenylpropanoids were identified in our work, with high concentrations, suggesting they may also be associated with the inhibition of nitric oxide production during the in vitro evaluation of anti-inflammatory potential. The results of the present study indicated a significant biological activity of the crude extract of *B*. *dracunculifolia* in reducing NO^•^ and O_2_^•−^ production in vitro.

Controlling excess free radicals in living organisms can also reduce oxidative stress on DNA, consequently helping to control inflammation and protect against tumor development. Studies related to the cytotoxic properties involving extracts of *B*. *dracunculifolia* are scarce [45], and there are only a few reports with extracts of green propolis [46]. Therefore, considering the antioxidant capacity of the extract from the aerial parts of *B*. *dracunculifolia* observed in this work, experiments were conducted to evaluate the antiproliferative capacity of this extract against four tumor cell lines: AGS (gastric adenocarcinoma), Caco-2 (colorectal adenocarcinoma), MCF-7 (breast adenocarcinoma), and NCI-H460 (lung carcinoma). The results of the antiproliferative activity are presented in Table 5. To the best of our knowledge, this is the first report of tests of *B. dracunculifolia* extracts against AGS, Caco-2, and NCI-H460 cell lines.

Additionally, the action of the crude extract was evaluated in a non-tumor cell line, VERO cells (African green monkey renal epithelial cells). VERO cells are a continuous cell line, meaning they can be propagated for a long time without losing their growth characteristics. It is important to note that this ability is maintained without the acquisition of tumor functions, unlike other primary cell lines that undergo limited passage numbers, allowing the derivation of several sublines and the storage of cells in cell banks [47].

Regarding the antiproliferative activity, the crude extract of *B. dracunculifolia* exhibited weak growth-inhibitory effects against all tested tumor cell lines, with GI_50_ values ranging from 165 to 257 µg/mL (Table 5), whereas the positive control, ellipticine, showed markedly higher potency (GI_50_ = 1.01–1.23 µg/mL). According to the classification proposed by Tauchen et al. [48], antiproliferative activity is considered strong when GI_50_ ≤ 60 µg/mL, moderate when GI_50_ ≤ 100 µg/mL, weak when GI_50_ > 100 µg/mL, and absent when GI_50_ > 500 µg/mL. Based on these criteria, the *B. dracunculifolia* crude extract can be classified as displaying weak antiproliferative activity.

The modest antiproliferative activity of the crude extract (GI_50_ = 165–257 μg/mL) likely reflects its complex phytochemical composition rather than the absence of bioactive constituents. UHPLC-HRMS analysis demonstrated that the extract is predominantly composed of phenolic compounds, particularly phenylpropanoids and flavonoids (Table 1), classes of metabolites widely recognized for their antioxidant, anti-inflammatory, and cytoprotective properties rather than for potent cytotoxic activity [49]. In particular, hydroxycinnamic acid derivatives, including caffeic acid and ferulic acid derivatives, generally act by modulating intracellular redox homeostasis and inflammatory pathways and typically exhibit relatively weak antiproliferative activity compared with classical cytotoxic phytochemicals [50].

The biological activity of crude botanical extracts cannot be interpreted as the sum of the effects of their individual constituents but rather as the result of synergistic, additive, and antagonistic interactions among chemically diverse metabolites [51,52]. In the present study, the predominance of phenylpropanoids and other antioxidant phenolics may have shifted the overall biological response toward the maintenance of cellular redox homeostasis, thereby masking the contribution of less abundant constituents with greater antiproliferative potential. Because modulation of intracellular redox signaling represents one of the mechanisms involved in apoptosis regulation, the high abundance of antioxidant metabolites may have attenuated oxidative stress-dependent cell death pathways, ultimately reducing the apparent cytotoxicity of the crude extract [53,54]. Although this hypothesis requires experimental confirmation, it provides a plausible explanation for the weak antiproliferative activity observed despite the chemical complexity of the extract.

The proposed interpretation is further supported by the cellular antioxidant assay, in which the crude extract inhibited intracellular oxidation by 81% (Table 4), demonstrating a pronounced ability to counteract oxidative stress in a cellular environment. Although this assay does not directly establish a cytoprotective effect, it indicates that the extract predominantly modulates cellular redox balance [55]. Given that oxidative stress represents one of the signaling mechanisms involved in apoptosis induction [56], the predominance of antioxidant phenolics may have favored the maintenance of redox homeostasis thereby reducing the contribution of oxidative stress-dependent cell death pathways to the overall biological response [53]. This mechanistic interpretation is consistent with the modest antiproliferative activity observed for the crude extract.

The selectivity index further reinforces this interpretation. SI values between 0.90 and 1.41 indicate essentially no preferential toxicity toward tumor cells, contrasting with the SI values (>10) generally considered desirable for candidate anticancer agents [57]. Notably, this low selectivity should not be interpreted as evidence of excessive toxicity toward normal cells. The crude extract exhibited a GI_50_ of 233.00 μg/mL in VERO cells, which is classified as non-cytotoxic according to Abdul Latif et al. [58]. Thus, the low SI values primarily reflect the limited antiproliferative potency against tumor cells rather than toxicity toward non-tumor cells. Collectively, these findings suggest that the crude extract exhibits low toxicity toward the non-tumor VERO cell line, although its selectivity as an antiproliferative agent remains limited under the experimental conditions evaluated.

Finally, although bioassay-guided fractionation was beyond the scope of the present study, this approach may enhance the apparent antiproliferative activity of the extract by enriching less abundant metabolites with greater cytotoxic potential while reducing the relative abundance of antioxidant phenolics. Such strategies are widely employed in natural product drug discovery to identify bioactive constituents that may be masked within chemically complex crude extracts [52,59]. Future studies should therefore investigate purified fractions and isolated compounds to determine whether the limited antiproliferative activity observed for the crude extract results from compositional dilution, antagonistic interactions among constituents, or both.

A study using components isolated from *B*. *dracunculifolia* leaves evaluated cytotoxicity against leukemia cells (L1210). The authors attributed the strong cytotoxic activity observed to phenolic monoterpenes, such as thymol, carvacrol, *p*-methoxythymol, and *p*-cymene-2,3-diol [45]. Several studies have shown that artepillin C, a specific metabolite of *B*. *dracunculifolia* and the main component of green propolis, is a potent antioxidant with chemopreventive activity against various cancers [60,61]. Furthermore, *p*-coumaric acid and its derivatives, baccharin and artepillin C, all important constituents of *B*. *dracunculifolia*, showed activity against breast and prostate cancer cells in vitro [46]. An in vitro study on the antigenotoxic and antimutagenic activities of *B. dracunculifolia* extract demonstrated protective effects on mammalian HTC cells at the same concentrations, without inducing any toxicity [27]. According to the authors, the antimutagenic effect is related to the extract’s ability to accept free radicals. Flora [62] suggests that the antimutagenic activity of phenolic compounds occurs primarily as a result of their ability to scavenge free radicals, which is one of the most important mechanisms of antimutagenicity and anticarcinogenicity.

The species *B*. *dracunculifolia* is found mainly in regions with poor soil and low-lying vegetation with little shade, which increases its exposure to the sun. To cope with such harsh environments, plants typically biosynthesize photoprotective metabolites that shield them from solar radiation and act as natural antioxidants [63]. This condition could justify the chemical profile of the aerial parts of *B*. *dracunculifolia*, represented mainly by phenolic and polyphenolic compounds in the present work, since these types of compounds have shown significant absorption in the ultraviolet A (UVA) and ultraviolet B (UVB) regions, due to the set of conjugated double bonds in their chemical structures. This characteristic makes them suitable as ingredients in cosmetic formulations for skin protection [64].

Therefore, considering the chemical profile of the *B*. *dracunculifolia* (Table 2), experiments were conducted to evaluate the photoprotective potential of this extract. According to the results presented in Table 6, SPF values ranged from 7.79 to 19.30, showing a concentration-dependent response. In addition to SPF, other photoprotective parameters were evaluated, including the UVA/UVB ratio, the UVA protection factor, and the critical wavelength (λc), as shown in Table 6.

The UVA/UVB ratio (Table 6) corresponds to the proportion between the area under the absorption curve in the UVA region (320–400 nm) and in the UVB region (290–320 nm) [66]. Ratios of 1.0 or higher indicate greater UVA protection (see Table in Section 3.11).

**Figure 1:**
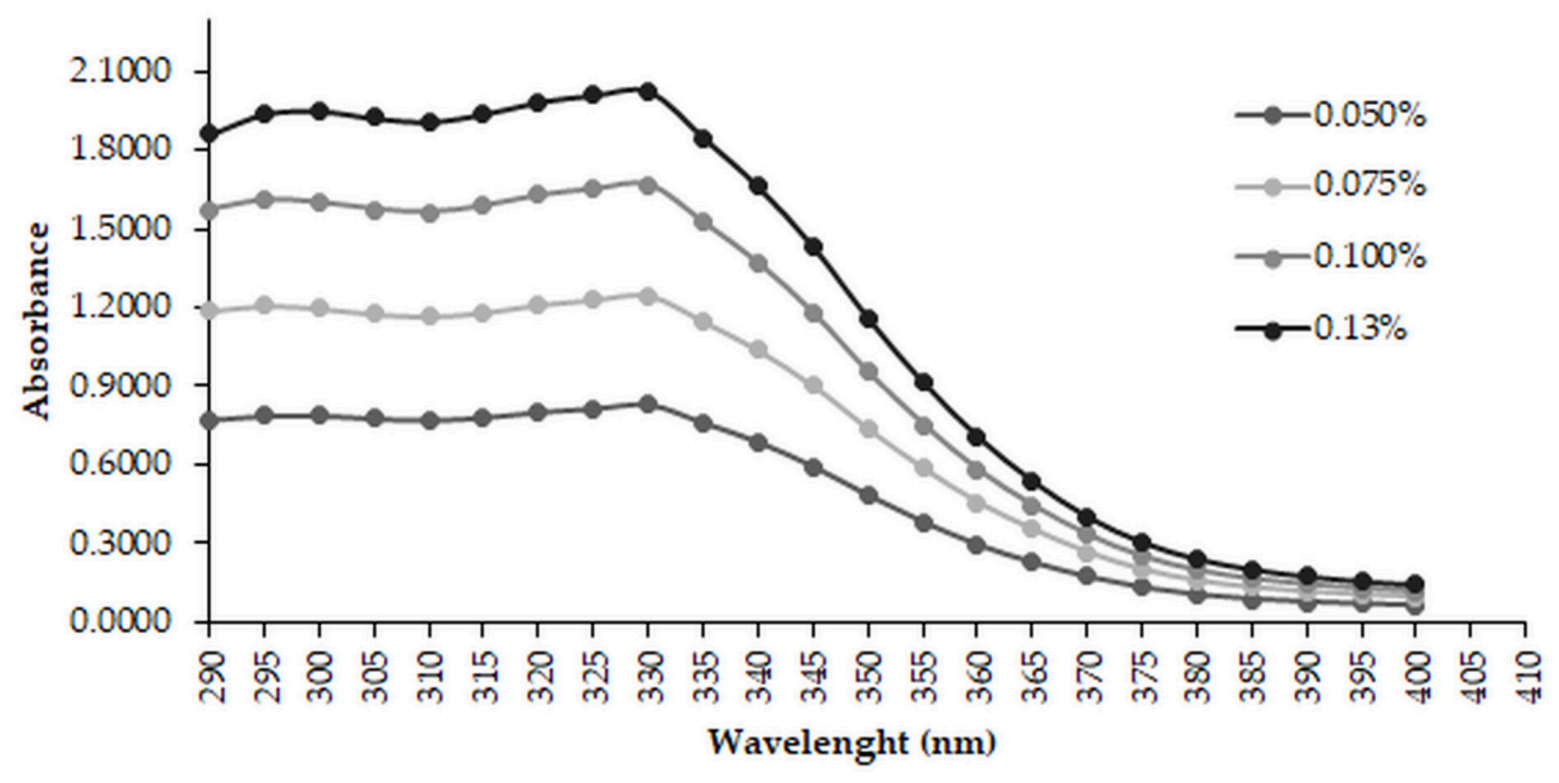
UV absorption spectra of the crude extract from the aerial parts of *Baccharis dracunculifolia* at concentrations between 0.050% to 0.125% over wavelengths from 290 to 400 nm.

As shown in Figure 1, the extract exhibited strong absorption throughout the UVB region and part of the UVA region, with absorbance gradually decreasing above 340 nm. The wavelength range near 340 nm is commonly associated with flavonoids and phenolic acids. The SPF values observed for the *B. dracunculifolia* extract are likely associated with the high concentrations of flavonoids and phenylpropanoids identified in Table 1. Both classes possess extended π-conjugated systems that act as natural chromophores, enabling efficient absorption of ultraviolet radiation. In particular, phenylpropanoids, especially hydroxycinnamic acid derivatives, play a central role in plant photoprotection by absorbing predominantly UVB radiation (280–320 nm). Therefore, the elevated content of these metabolites may explain, at least in part, the absorption observed across the UVB region and part of the UVA range, as well as the SPF values obtained for the extract [67]. According to Brazilian regulatory standards, RDC nº 629 [63], SPF values ≥ 6.0 are required for consideration in the development of sunscreens. Based on these criteria, the results support further investigation of *B*. *dracunculifolia* extract as a source of photoprotective compounds.

Vilas-Boas et al. [21] analyzed the antioxidant and photoprotective potential of an optimized extract from *B*. *dracunculifolia* leaves rich in phenolic substances. According to the authors, based on in silico analysis of individual compounds, all showed UV absorption within the desired range for photoprotection. According to them, it was observed that phenylpropanoids (cinnamic, *p*-coumaric, ferulic, and caffeic acids; allyl 3-prenylcinnamate, artepillin C) exhibited maximum absorption in the UVB or UVA I range, while flavonoids (kaempferol, quercetin, and rutin) showed two peaks, one in the UVB range and the other, more intense, in the UVA II range. In this way, the combination of these compounds would provide broad-spectrum absorption, supporting the use of the extract rather than isolated molecules, because compounds with distinct absorption maxima may complement one another when present in the same extract [21]. The described photoprotective potential is based on preliminary physicochemical data. Further studies in biological models are needed to expand knowledge about the photoprotective activity of *B. dracunculifolia*.

The chemical characterization revealed several metabolites previously associated with antioxidant, anti-inflammatory, antiproliferative, and photoprotective activities. To provide additional context for the experimental findings, the identified compounds were subsequently evaluated using complementary computational approaches, including PASS prediction, SwissTargetPrediction, GO enrichment analysis, ADMET profiling, and toxicity prediction.

First, the biological potential of each of the identified secondary metabolites was investigated using PASS analysis to predict which of them could be associated with the properties observed in the in vitro activity tests evaluated in this work: antioxidants and correlates (free radical scavenger, lipid peroxidase inhibitor), anti-inflammatory, and anticancer (apoptosis agonist, antineoplastic, anticancer, chemopreventive). The results of the biological potential prediction for these specific activities are presented in Table 7, where only values with Pa > 0.3 were considered.

All metabolites showed predicted antioxidant, free radical-scavenging potential, and lipid peroxidase inhibition capacities, with activity probabilities ranging from 31.0% to 98.8%, except for nicotinic acid and caffeine. Overall, chlorogenic acid and flavonoids in general were the compounds most likely to exhibit antioxidant capacity, probably due to their polyphenolic structures. Thus, these predictions were consistent with the antioxidant activity observed experimentally and detected using methodologies that express distinct mechanisms of action, since these compounds exhibit antioxidant activity at different levels due to resonance or better charge stabilization. Together, this suggests that the combination of different antioxidants promotes a synergistic action.

Regarding anti-inflammatory activity, all compounds except nicotinic acid are predicted to exhibit this property, with coumarin, quinic acid, and salicylic acid, as well as phenylpropanoids and flavonoids in general, being the most active. On the other hand, phenolic acids, specifically, showed high intestinal anti-inflammatory activity. In general, these in silico predictions of the anti-inflammatory activity of the metabolites identified in the extract were consistent with the inhibition of nitric oxide production observed in the in vitro test using RAW 264.7 macrophage cells.

Beyond these predicted activities, PASS analysis also indicated cytotoxic-related properties among the identified compounds, including anticancer, apoptosis agonist, antineoplastic, and chemopreventive potential, with flavonoids consistently the most active. Flavonoids showed the highest probabilities for anticancer and chemopreventive activities and were also associated as apoptosis agonists. For antineoplastic activity, quinic acid and flavonoids showed excellent probabilities, while phenylpropanoids were moderately active; flavonoids, however, showed low predicted antineoplastic activity against breast, small-cell-lung and lung cancer cells, and chrysin and luteolin showed similarly low activity against colorectal and colon cancer cells. Based on these predictions, we suggest that phenylpropanoids and flavonoids contribute to the antiproliferative activity observed in the in vitro experiments.

In parallel with the biological activity predictions, potential enzyme and protein targets in *Homo sapiens* were predicted for all secondary metabolites identified using the SwissTargetPrediction platform [68,69]. The prenylated phenylpropanoids baccharin, drupanin, and artepillin C—recognized as important bioactive metabolites of green propolis and *B. dracunculifolia* [1]—were also included in the in silico analyses, although they were not quantified in this study due to the unavailability of authentic standards. Most analyzed compounds (~66%), including these three, showed no relevant predicted affinity for molecular targets associated with the biological activities evaluated here. In contrast, nine compounds exhibited predicted interactions above 10% for molecular targets previously associated with oxidative stress, inflammation, cell proliferation, and UV response pathways (Table 8), with the flavonoids chrysin, luteolin, kaempferol, and quercetin stood out for their broader predicted target interactions profiles.

To provide functional interpretation of the predicted molecular targets, GO enrichment analysis was conducted using the PANTHER classification system. Representative enriched GO biological processes are presented in Table 9. The enriched terms were mainly associated with oxidative stress and ROS regulation, inflammatory and prostaglandin-related processes, apoptosis/cell-cycle signaling, and cellular responses to UV radiation. These findings are consistent with the antioxidant, anti-inflammatory, antiproliferative, and photoprotective activities experimentally observed for the extract.

GO enrichment analysis highlighted biological processes related to oxidative stress, ROS metabolism, and cellular responses to hydrogen peroxide, which is consistent with the experimentally observed antioxidant activity of the extract (Table 9). Predicted targets, including NOX4 (NADPH oxidase 4), XDH/XO (xanthine dehydrogenase/oxidase), and MAOA/MAOB (monoamine oxidases A and B), are involved in intracellular ROS production and redox imbalance. These enzymes have been associated with oxidative stress, inflammatory disorders, and cancer-related processes [70,71,72]. Among the metabolites identified in the extract, quercetin, kaempferol, and luteolin showed higher predicted probabilities of interaction with NOX4 and MAOA. Chrysin also showed higher predicted interaction probabilities for XDH/XO-associated targets. Although these predictions do not confirm the molecular mechanisms involved, they reinforce the possibility that flavonoids in the *B. dracunculifolia* extract contribute, at least in part, to the antioxidant effects observed in vitro through interactions with multiple redox-related targets.

GO enrichment analysis further identified biological processes associated with inflammatory response and prostaglandin/prostanoid metabolism, consistent with the experimentally observed nitric oxide-inhibition activity of the extract (Table 9). The targets PTGS2 (cyclooxygenase-2), CXCR1 (interleukin-8 receptor A), MMP9 (matrix metalloproteinase 9), F2 (thrombin), and SYK (tyrosine-protein kinase) are associated with inflammatory signaling, extracellular matrix remodeling, angiogenesis, and tumor progression. PTGS2 is involved in prostaglandin biosynthesis, while CXCR1 and MMP9 are related to cell recruitment during the inflammatory response and in extracellular matrix degradation [73,74]. F2 and SYK also participate in signaling events associated with inflammation and immune responses [75]. Quercetin, kaempferol, and luteolin showed higher predicted interaction probabilities with several of these targets. Naringenin, chrysin, and morin exhibited lower affinities. Although these predictions require experimental confirmation, the results suggest that flavonoids present in the extract may be involved in the modulation of inflammatory pathways through interactions with different molecular targets.

The antiproliferative activity observed for the extract may also be associated with the interaction of some identified flavonoids with targets involved in cell survival, apoptosis, and cell-cycle regulation. This interpretation agrees with the GO enrichment results, which revealed biological processes related to apoptotic regulation and G2/M cell-cycle transition (Table 9). Among the molecular targets identified, EGFR (epidermal growth factor receptor erbB1), MET (hepatocyte growth factor receptor), SRC (tyrosine-protein kinase SRC), AKT1 (serine/threonine-protein kinase), PLK1 (Serine/threonine-protein kinase PLK1), and cyclin-dependent kinases (CDKs) are associated with signaling pathways linked to tumor progression and uncontrolled cell proliferation [76,77]. Quercetin showed the highest predicted interaction probabilities for several of these targets, while kaempferol and luteolin presented more moderate profiles. Chrysin also demonstrated relevant predicted affinity for some CDK-related targets. The computational predictions are consistent with the antiproliferative effects observed experimentally in tumor cell lines and support the hypothesis that flavonoids contribute to the biological activity of the extract.

Although no molecular targets associated with photoprotective activity were identified in the target prediction analysis, the GO enrichment results revealed biological processes related to cellular responses to UV radiation and DNA damage (Table 9). Terms such as response to UV-A, cellular response to UV-A, and DNA damage response suggest that metabolites identified in the *B. dracunculifolia* extract may be associated with pathways involved in cellular protection against UV-induced stress [78]. These findings are consistent with the photoprotective activity experimentally observed for the extract, including its sun protection factor and UVA/UVB ratio. Given the predominance of phenylpropanoids and flavonoids in the extract, it is possible that part of this effect is due to their ability to absorb UV radiation and modulate oxidative stress-related cellular responses [79].

Overall, the in silico analyses provide a framework for interpreting how different metabolites identified in the extract may contribute to the biological activities observed experimentally through interactions with multiple molecular targets and biological processes related to oxidative stress, inflammation, cell proliferation, and UV response. Interestingly, some compounds with higher predicted interaction probabilities were detected at relatively low abundance in the extract, highlighting that biological activity may not depend exclusively on compound abundance. At the same time, the absence of strong predicted interactions for other metabolites does not exclude their contribution to the biological effects of the extract. Several abundant compounds identified in the extract, including morin [80], naringenin [81,82], coumaric acid [83,84], ferulic acid [85], caffeic acid [86], chlorogenic acid, and rutin [87,88], have previously been associated with antioxidant, anti-inflammatory, and anticancer activities.

In addition to the predicted biological activities and molecular targets, the pharmacokinetic properties of the identified metabolites were also evaluated through in silico approaches. The pharmacokinetic properties of the identified metabolites were estimated using SwissADME, considering parameters related to absorption, distribution, metabolism, and excretion (ADME) [89].

The pharmacokinetic profiles (ADME) of all phytocompounds identified were estimated from their structural and physicochemical data to evaluate characteristics relevant to oral bioavailability and drug-likeness. The main results of this analysis are shown in Table 10.

A fundamental principle for determining the pharmacokinetics of natural or synthetic compounds as potential drug candidates is Lipinski’s Rule of Five (RO5) [90] commonly used as a preliminary indicator of oral drug-likeness. Most of the phytoconstituents analyzed met drug-likeness criteria, except for rutin, which violated Lipinski’s rule in three ways: Molecular mass = 610.52 g/mol, more than 10 hydrogen bond acceptors, and more than 5 hydrogen donors.

**Figure 2:**
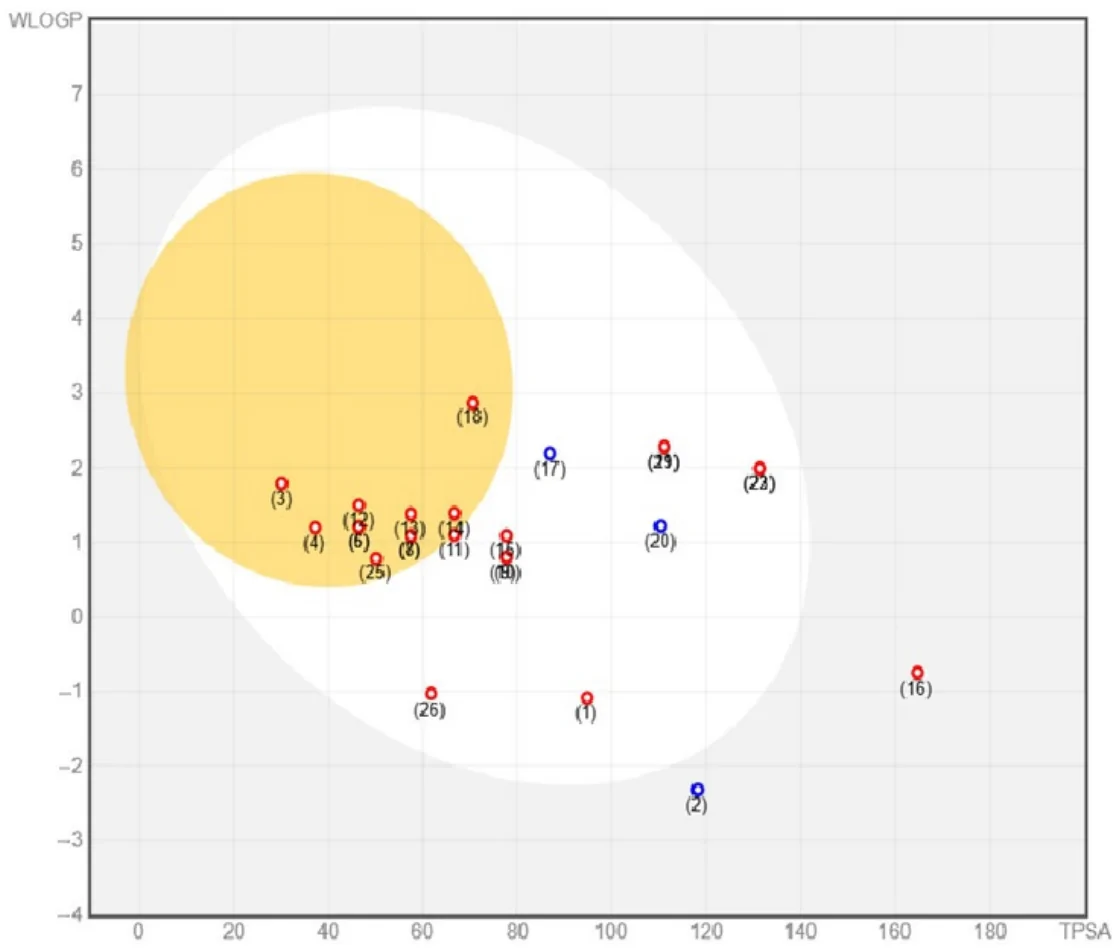
Boiled-EGG model for evaluating human intestinal absorption (HIA), blood–brain barrier (BBB) permeation of P-glycoprotein (P-gp) substrate permeated through the blood–brain barrier (BBB) of compounds identified in the extract of *B*. *dracunculifolia*. [WLOGP: lipophilicity; TPSA: topological area of the polar surface. The white region is for high probability of passive absorption by the gastrointestinal tract, and the yellow region (yolk) is for high probability of brain penetration. Compounds in the grey region are predicted as not absorbed and not brain penetrant. The points are colored in blue if predicted as actively effluxed by P-gp (PGP+) and in red if predicted as non-substrate of P-gp (PGP−)]. (1) Malic acid; (2) Quinic acid; (3) Coumarin; (4) *p*-Hydroxybenzaldheyde; (5) Vanillin; (6) Isovanillin; (7) Salicylic acid; (8); *p*-Hydroxybenzoic acid; (9) Protocatechuic acid; (10) α-Resorcylic acid; (11) Vanillic acid; (12) Coniferaldehyde (13) *p*-Coumaric acid; (14) Ferulic acid; (15) Caffeic acid; (16) Chlorogenic acid; (17) Naringenin; (18) Chrysin; (19) Luteolin; (20) (-)-Epicatechin; (21) Kaempferol; (22) Morin; (23) Quercetin; (24) Rutin; (25) Nicotinic acid; (26) Caffeine.

The BOILED-Egg (Brain or IntestineL EstimateD) predictive permeation model provides a method for predicting human gastrointestinal absorption (HIA) and brain access of small molecules [89]. In this model, the egg white indicates a high probability of passive gastrointestinal absorption, while the yolk indicates a high probability of cerebral penetration [91]. Gastrointestinal absorption and blood–brain barrier permeability were further evaluated using the BOILED-Egg model (Figure 2). According to the pharmacokinetic profile calculated from the physicochemical parameters of the metabolites, most compounds demonstrated optimal absorption, as indicated by their high human gastrointestinal absorption (HIA) values. In contrast, their solubility profiles suggest efficient gastrointestinal absorption. Only rutin, quinic, and chlorogenic acids showed low passive absorption, limiting their absorption in the GI tract due to their high polarity and, in the case of rutin, its high TPSA value. Except for these three constituents, the others showed good potential for oral administration, indicating favorable bioavailability. In terms of distribution, blood–brain barrier (BBB) permeability analysis indicated that ten compounds showed a probability of cerebral penetration, namely coumarin, *p*-hydroxybenzaldehyde, vanillin, isovanillin, coniferaldehyde, chrysin, and salicylic, *p*-hydroxybenzoic, *p*-coumaric, ferulic, and nicotinic acids. These results indicate the potential of some metabolites to reach central nervous system tissues following systemic administration.

P-glycoprotein (P-gp) substrate prediction was also evaluated because this transporter influences intestinal absorption, blood–brain barrier penetration, and multidrug resistance mechanisms [92]. Moreover, it is important to note that P-gp is overexpressed in some tumor cells, leading to the development of multidrug-resistant cancers. Naringenin and (-)-epicatechin were predicted to be P-gp substrates, which means that they may be subject to efflux by P-gp transporters, potentially decreasing their effectiveness by reducing cellular uptake.

Similar pharmacokinetic predictions have been reported for constituents of *B. dracunculifolia*. Using SwissADME, Cappellucci et al. [93] identified compounds with favorable gastrointestinal absorption and, in some cases, predicted blood–brain barrier permeability. Their findings also indicated that only a subset of metabolites behaved as P-gp substrates, which is consistent with the limited number of P-gp substrates identified in the present study.

**Figure 3:**
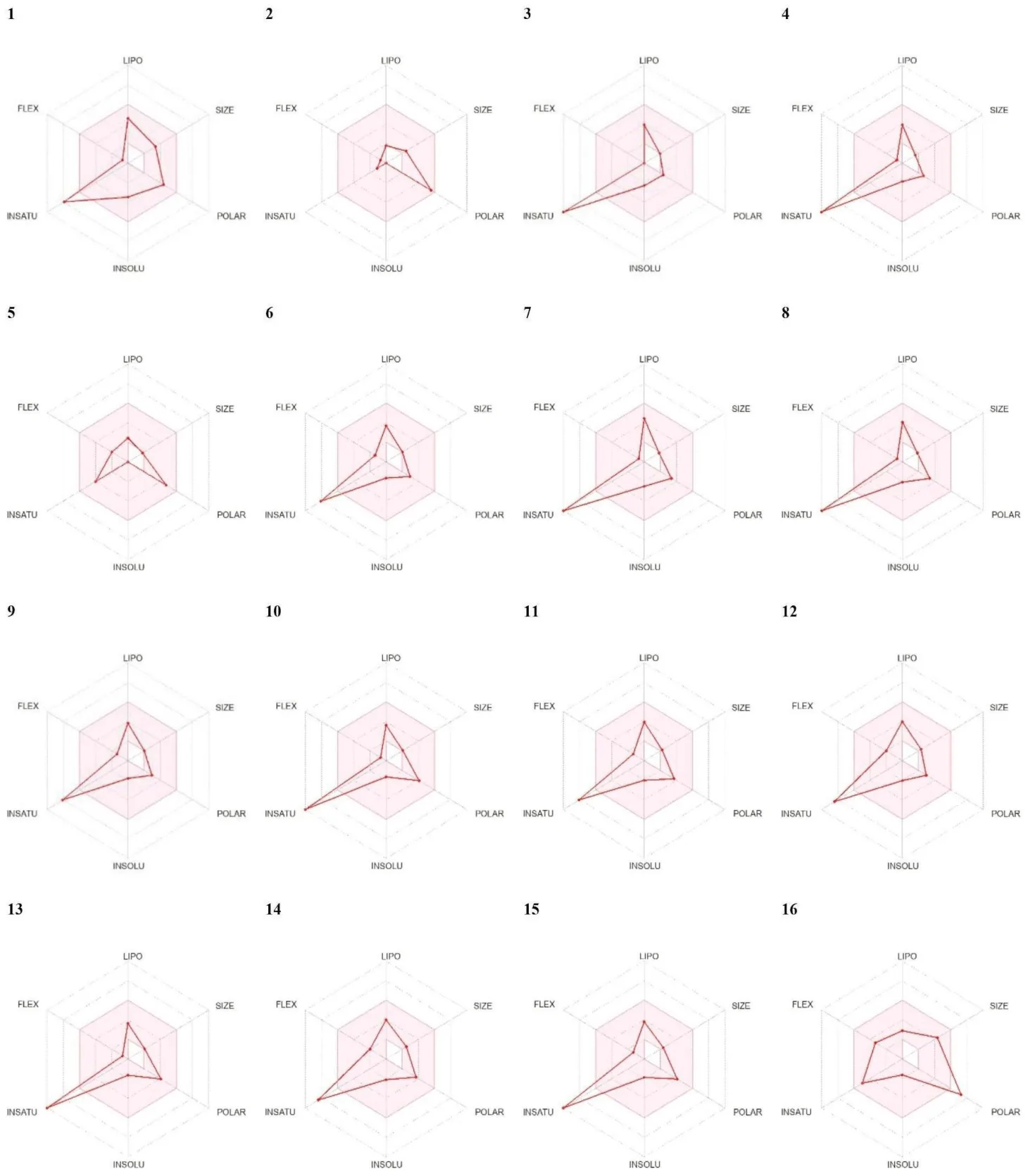

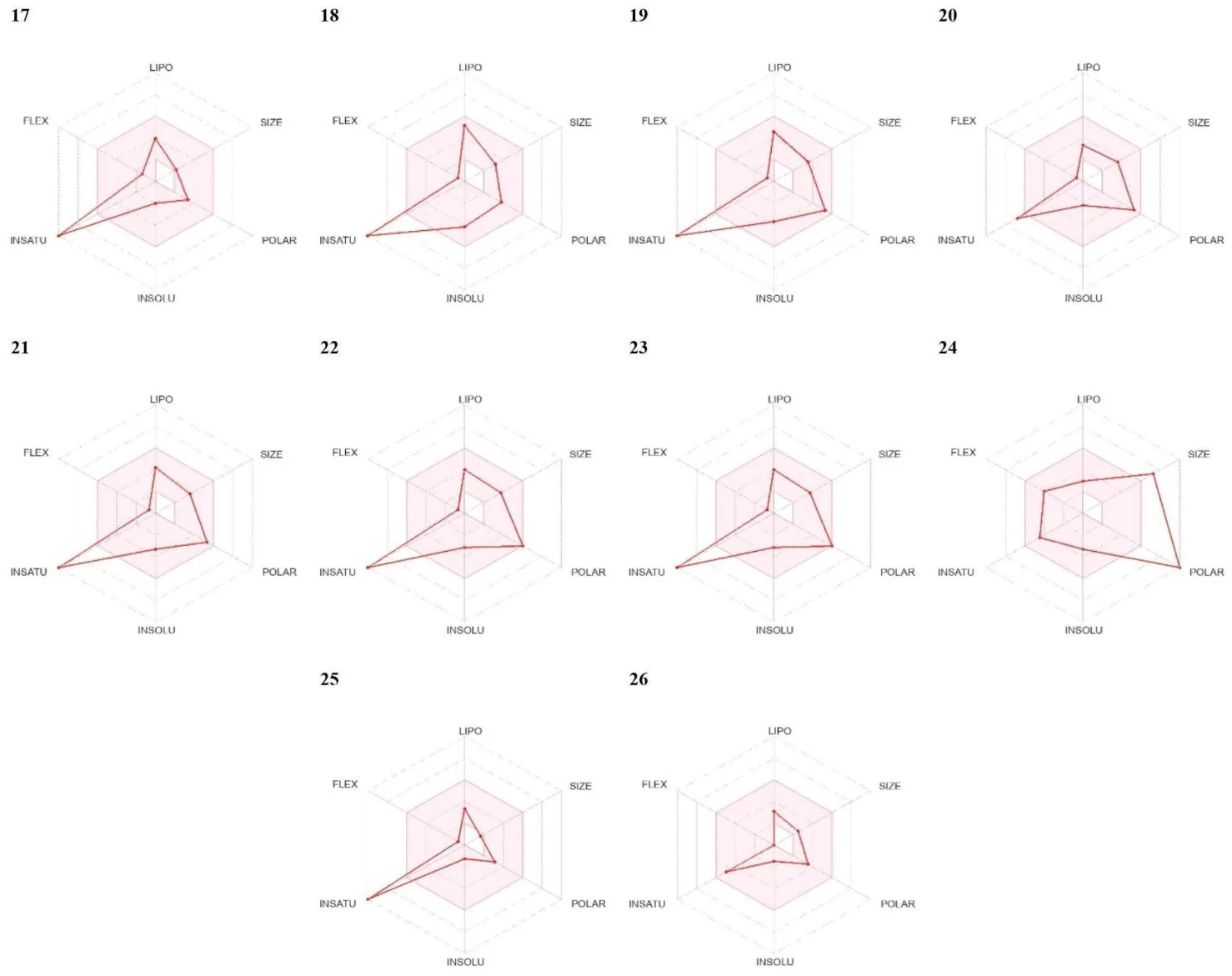
Oral bioavailability analysis of secondary metabolites identified in the extract of the aerial parts of *B*. *dracunculifolia*. [The colorful zone is the ideal physical space for bioavailability in the mouth. LIPO (lipophilicity): −0.7 < XLOGP3 < +5.0; SIZE: 150 g/mol < MW < 500 g/mol; POLAR (polarity): 20 Å^2^ < TPSA < 130 Å^2^; INSATU (insaturation): 0.15 < Fraction Csp^3^ < 1; FLEX (flexibility): 0 ≤ number of rotables bonds < 9; INSOLU (insolubility): −6 < Log S (ESOL) < 0] (1) Malic acid; (2) Quinic acid; (3) Coumarin; (4) *p*-Hydroxybenzaldheyde; (5) Vanillin; (6) Isovanillin; (7) Salicylic acid; (8); *p*-Hydroxybenzoic acid; (9) Protocatechuic acid; (10) α-resorcylic acid; (11) Vanillic acid; (12) Coniferaldehyde (13) *p*-Coumaric acid; (14) Ferulic acid; (15) Caffeic acid; (16) Chlorogenic acid; (17) Naringenin; (18) Chrysin; (19) Luteolin; (20) (-)-Epicatechin; (21) Kaempferol; (22) Morin; (23) Quercetin; (24) Rutin; (25) Nicotinic acid; (26) Caffeine.

A drug’s ability to penetrate membranes for transport throughout the body is highly correlated with its physicochemical properties [91]. Bioavailability radar analysis was used to evaluate six physicochemical properties associated with oral drug-likeness (Figure 3). Taken together, these analyses provide information on the pharmacokinetic suitability of the identified metabolites. According to the bioavailability radars presented in Figure 3, except for caffeine and the organic acids malic and quinic, all other phytocompounds showed less favorable predicted oral bioavailability, mainly due to their high unsaturation (sp^3^ carbon fraction = 0.0). Furthermore, morin and quercetin showed polarity close to the limit (TPSA = 131.36 Å^2^), while chlorogenic acid and rutin showed polarities above the expected limit (TPSA = 164.75 Å^2^ and 269.43 Å^2^, respectively), compromising the oral bioavailability of these compounds.

The bioavailability score aims to predict the likelihood of a compound having at least 10% oral bioavailability in rats or measurable permeability in CaCo-2 cells [89]. The high polarity of chlorogenic acid and rutin, in addition to their high molecular weight, were determining factors in justifying the low bioavailability score (0.11 and 0.17, respectively) of these two phytocompounds, as well as the low gastrointestinal absorption shown previously in Figure 2. According to Table 10, the aglycone flavonoids and other compounds with moderate bioavailability scores (~0.55) showed good oral absorption. On the other hand, simpler phenolic acids, such as salicylic, *p*-hydroxybenzoic, vanillic, *p*-coumaric, ferulic, and nicotinic acids, showed the highest bioavailability scores (0.85) (Table 10). In the case of flavonoids, the predicted pharmacokinetic profiles were more favorable for aglycones than for their glycosylated counterparts.

Skin permeability is a crucial parameter in the development of new drugs or cosmetics. The in silico prediction of the permeability coefficient (Kp) for the transport of compounds through mammalian epidermis is based on the linear model of Potts and Guy [94] and is a useful tool for estimating topical or transdermal absorption. Generally, values close to −4 up to −6 suggest good permeability, while values below −8 indicate low permeability. According to these criteria, among the compounds identified in the extract of *B*. *dracunculifolia*, chrysin and salicylic acid were the metabolites with the best skin permeability values, while malic, chlorogenic, and quinic acids, as well as rutin, should present low permeability through the epidermis. The other phytocomponents evaluated suggest moderate skin permeability (Table 10). These predictions are particularly relevant considering the photoprotective activity observed for the extract and may be useful for future studies exploring topical or sunscreen formulations.

Toxicity profiles of the identified metabolites were estimated using the ProTox-III platform, which predicts acute toxicity, organ toxicity, and toxicological endpoints based on molecular structure [95,96,97]. The online server ProTox-III [97] was used to interpret and analyze the results of the toxicity class and probability of oral toxicity, organ toxicity, and toxicological outcomes (carcinogenicity, immunotoxicity, mutagenicity, and cytotoxicity) of the metabolites identified in the extract of *B*. *dracunculifolia*, whose results are detailed in Table 11.

Regarding the probability of oral toxicity, three compounds were classified as Class III: coumarin, quercetin, and caffeine. The remaining compounds are in Classes IV–VI (Table 11).

Concerning organ toxicity, except for caffeine and *p*-hydroxybenzaldehyde, all other compounds showed probabilities of nephrotoxicity, with salicylic acid and rutin standing out. Flavonoids showed a probability of respiratory toxicity, with naringenin, luteolin, kaempferol, morin, and quercetin standing above 82%. Nicotinic acid was hepatotoxic with an 80% probability, and caffeine had a 96% probability of being neurotoxic. None of the identified compounds showed a probability of being cardiotoxic (Table 11).

Regarding the toxicological outcomes (carcinogenicity, immunotoxicity, mutagenicity, and cytotoxicity) of the identified compounds (Table 10), coumarin showed the highest probability of carcinogenicity, followed by caffeic acid. Higher probabilities of neurotoxicity were predicted for rutin and for ferulic and chlorogenic acids.

Overall, the computational analyses were consistent with the antioxidant, anti-inflammatory, antiproliferative, and photoprotective activities observed experimentally and provided a complementary framework for interpreting the potential contribution of individual metabolites. Although in silico computational studies provide useful initial insights into a metabolite’s potential as a drug, they have limitations. They are essentially predictive and cannot account for all biological factors. Therefore, it is crucial to recognize that experimental validation, including in vitro and in vivo studies, will be necessary to validate the biological relevance of the in silico predictions and establish the efficacy, bioavailability, and safety of the evaluated metabolites. This research will be necessary in the future to understand the therapeutic importance of these phytoconstituents and to guide the selection of the best candidates for the development of pharmaceutical or photoprotective products.

Although the identified metabolites exhibited heterogeneous pharmacokinetic and toxicity profiles, several compounds showed favorable predicted ADMET characteristics, including gastrointestinal absorption and acceptable toxicity estimates. These properties may justify their prioritization for future pharmacological and toxicological investigations.

Finally, the presence of phenolic compounds identified in the aerial parts of *B. dracunculifolia*, aligned with the in vitro and in silico assays presented, provide a basis for future studies focused on the isolation, characterization, and biological evaluation of individual metabolites. Such investigations may help clarify their contribution to the biological activities of the extract and identify compounds with greater potential for pharmaceutical or photoprotective applications.

## 3. Materials and Methods

### 3.1. Plant Material

The aerial parts of *B. dracunculifolia*, including flower buds and leaves, were collected at coordinates 23°39′49.7″ S and 53°18′18.0″ W, along the Boiadeira road, between the municipalities of Maria Helena and Umuarama, in the state of Paraná, Brazil. The collection took place in the morning, between 7 and 8 am, on 7 May 2024, which corresponds to the early stages of bud emergence for this species [6]. Dr. Ezilda Jacomassi conducted the botanical identification, and a specimen has been deposited in the University of Paraná Herbarium under the number 375. Access to the botanical source was authorized and licensed by CGen/SisGen, registered under the code AB29E78.

### 3.2. Obtaining the Crude Extract

The aerial parts, composed of leaves and flower buds, were collected and dried on plant drying mats at room temperature (32 °C). After drying, the plant material was pulverized in a Willye-type knife mill (TE 650) (Tecnal Equipamentos Científicos, São Paulo, Brazil), and the particle size distribution was determined at 850 µm. The resulting powder (230 g) was subjected to a dynamic maceration process with solvent renewal using 96% (*v*/*v*) ethyl alcohol [98]. Solvent renewal was carried out until the plant material was exhausted, as described in the Brazilian Pharmacopoeia [1]. Subsequently, the extract was concentrated in a rotary evaporator (Tecnal TE-211 model) (Tecnal Equipamentos Científicos, São Paulo, Brazil) at 40 °C, until the crude extract of the aerial parts of *B. dracunculifolia* (70.0 g) was obtained, yielding 30.46%, which was then packaged in an amber bottle and stored at −18 °C.

### 3.3. Materials and Reagents

HPLC-grade methanol and the analytical standards—malic acid, fumaric acid, quinic acid, coumarin, catechol, hydroxybenzaldehyde, vanillin, isovanillin, syringaldehyde, sinapaldehyde, salicylic acid, *p*-hydroxybenzoic acid, protocatechuic acid, α-resorcylic acid, vanillic acid, gallic acid, sinapic acid, syringic acid, coniferyl aldehyde, *p*-coumaric acid, caffeic acid, ferulic acid, chlorogenic acid, naringin, naringenin, chrysin, baicalin, catechin, (-)-epicatechin, luteolin, kaempferol, quercetin, morin, rutin, theobromine, nicotinic acid, caffeine, and syringaldazine—were purchased from Sigma-Aldrich (St Louis, MO, USA).

### 3.4. Chemical Identification by UHPLC-MS/MS

The crude extract from the aerial parts of *B. dracunculifolia* was analyzed by ultra-high performance liquid chromatography with tandem mass spectrometry (UHPLC-MS/MS) (Shimadzu^®^ model 8050 MS and NexeraR X2 HPLC, Kyoto, Japan). The analysis used an electrospray ionization (ESI) source and was conducted in both negative and positive ion modes, utilizing multiple reaction monitoring (MRM) for detection. The MS/MS detector operated in scan mode for 15 min. Collision energies were −15 V for the positive mode and 30 V for the negative mode. An interface voltage of 3 kV, a current of 7 µA, a temperature of 300 °C, and gas flow rates of 3 L/min for atomization and 10 L/min for drying were used. Argon served as the collision gas at a maximum pressure of 20 mPa. Chromatographic separation was performed with a C18 column (5 µm, 150 × 4.6 mm, Shimadzu^®^). Mobile phases consisted of Milli-Q water containing 0.1% formic acid (A) and MS-grade methanol (Merck^®^ Darmstadt, Germany). The method employed a linear gradient: 1–9 min (20% B), 10–15 min (40% B), and 16–30 min (10% B), at a flow rate of 0.5 mL/min, and a temperature of 40 °C. The sample was filtered through a hydrophobic polyvinylidene fluoride (PVDF) membrane with a 0.45 µm pore size and 25 mm diameter, with an injection volume of 1 µL. External standards (described in Section 3.3) were used to obtain the calibration curves with linear fit, and, regression coefficients > 0.98. The limits of quantification ranged from 10 to 250 µg L^−1^, while the limit of detection was 1 µg L^−1^.

### 3.5. Evaluation of the Total Phenol Content (TP) of the Crude Extracts of B. dracunculifolia Aerial Parts

The total phenol (TP) content in the CE of the aerial parts of *B. dracunculifolia* was measured by visible spectroscopy using the Folin–Ciocalteu method [24,25]. The extract samples were diluted in methanol to 1.0 mg/mL. The reagent solution included 155 μL of Folin–Ciocalteu reagent and 125 μL of sodium carbonate solution, followed by adding 20 μL of the diluted sample to each well of the microplate.

The mixture was left to stand in the dark for 60 min, then read at 760 nm on the SpectraMax Plus384 Microplate Reader in triplicate. The calibration curve was obtained using seven dilutions of gallic acid (0–100 µ/mL). The equation of the calibration curve obtained by linear regression is (Equation (1), R^2^ = 0.9997)

A = 0.0196 C − 0.031(1)

where A represents the measured absorbance, C the concentration of gallic acid equivalents, and R^2^ represents the coefficient of determination for the multiple regression. The results were expressed as µg of gallic acid equivalents (GAE) per mg of sample.

### 3.6. Evaluation of the Flavonoid Content of the Crude Extracts of B. dracunculifolia Aerial Parts

The total flavonoid content was measured using the aluminum chloride colorimetric method according to [25]. CEs from the aerial parts of *B. dracunculifolia* were dissolved in methanol to achieve concentrations of 1.00, 0.75, 0.50, and 0.25 mg/mL. An aliquot of 0.5 mL of extract was combined with 0.5 mL of 2% (*w*/*v*) aluminum chloride solution in methanol and kept at room temperature for 10 min. The absorbance change was measured at 425 nm. The aluminum chloride solution served as an analytical control. The flavonoid concentration was determined using the quercetin standard curve (5–40 µg/mL). Results were expressed as µg quercetin equivalents (EQ) per mg of extract.

### 3.7. Antioxidant Activities of B. dracunculifolia Crude Extract

#### 3.7.1. Scavenging of 2.2-Diphenyl-1-picrylhydrazyl (DPPH^•^) Free Radicals

The methodology described by [24,25] was used to determine the free radical scavenging capacity of DPPH. An aliquot of 10 µL of different concentrations of the CEs from the aerial parts of *B. dracunculifolia*, at concentrations (1.0, 0.75, 0.5, and 0.25 mg/mL), and 290 µL of methanolic DPPH^•^ solution (60 µM) were added to a SpectraMax Plus 384 Microplate Reader (Molecular Devices, San Jose, CA, USA) and kept for 30 min. The absorbance was measured at 515 nm. For the negative control, 10 µL of methanol was used with the DPPH^•^ solution (60 µM). The total antioxidant capacity of the extracts and fractions was calculated using a standard solution of quercetin (60 µM) as a 100% reference. Based on the correlation between absorbance and antioxidant concentration, the concentration needed to reduce free radical levels by 50% (IC_50_) was determined.

#### 3.7.2. Ferric Reducing Antioxidant Power (FRAP)

For the preparation of the FRAP reagent, 25 mL of acetate buffer (0.3 M), 2.5 mL of aqueous solution of 2,4,6-Tris(2-pyridyl)-striazine (TPTZ—10 mM), 2.5 mL of aqueous solution of ferric chloride (20 mM) and 3 mL of distilled water were combined. 25 mL of acetate buffer (0.3 M), 2.5 mL of aqueous solution of 2,4,6-Tris (2-pyridyl)-striazine (TPTZ—10 mM), 2.5 mL of aqueous solution of ferric chloride (20 mM), and 3 mL of distilled water were combined. The reagent solution consisted of 10 μL of the CEs from the aerial parts of *B. dracunculifolia* at concentrations of 1.00, 0.75, 0.50, and 0.25 mg mL^−1^ and 290 μL of the FRAP reagent in each well of the microplate. The mixture was placed in the SpectraMax Plus384 Microplate Reader and kept at 37 °C for 30 min. Absorbance was read at 595 nm. The percentage of antioxidant activity was calculated using a standard curve of ferrous sulfate (0–2000 μM). Antioxidant activity was expressed as μM ferrous sulfate per mg of sample [24,25].

#### 3.7.3. ABTS^•+^ Radical Method (2,2′Azinobis-(3ethylbenzthiazoline-6-sulfonic Acid))

To determine antioxidant activity using the ABTS^•+^ radical method, the methodology described by [25] was used. Initially, the ABTS^•+^ radical was generated by reacting 5.0 mL of 7 mM ABTS with 88 μL of 140 mM potassium persulfate, which were incubated at room temperature in the dark for 16 h. After this time, 1 mL of the solution was diluted in ethanol until an absorbance of 0.70 ± 0.05 nm at 734 nm was obtained. Microplates with 96 wells were used for the analysis, with 10 μL of the extracts (at concentrations of 1.0, 0.75, 0.50, and 0.25 mg mL^−1^) and 290 μL of the ABTS^•+^ radical solution added to each well. The mixture was placed in the SpectraMax Plus384 Microplate Reader, and the absorbance was determined after 30 min of reaction. The synthetic antioxidant Trolox was used as a standard solution at concentrations of 100, 500, 1000, 1500, and 2000 μM in ethanol. All the readings were carried out in triplicate, and the results were expressed as mM of Trolox per gram of *B. dracunculifolia* aerial part crude extract.

### 3.8. Cellular Antioxidant Activity

To assess cellular antioxidant activity (CAA), the procedure followed was that previously described by de La Fuente et al. [88]. Briefly, RAW 246.7 murine macrophages, commercially acquired from the European Collection of Authenticated Cell Cultures (ECACC) (Salisbury, UK), were routinely maintained with Dulbecco’s Modified Eagle Medium (DMEM) (HyClone, Logan, UT, USA) supplemented with L-glutamine (2 mM), penicillin (100 U mL^−1^), streptomycin (100 μg mL^−1^), fetal bovine serum (10%), and non-essential amino acids (2 mM) in T75 culture flasks at 37 °C in a humidified air incubator with 5% CO_2_ (Heal Force CO_2_ Incubator; Shanghai Lishen Scientific Equipment Co., Ltd., Shanghai, China).

The crude extracts were dissolved in H_2_O:DMSO (50/50, *v*/*v*) and H_2_O, respectively, to a final concentration of 8 mg mL^−1^. This solution was further diluted with 2′,7′-dichlorofluorescin (DCFH) prepared in ethanol, then diluted with HBSS (50 μM) to obtain the final test concentrations, ranging from 500 to 2000 μg mL^−1^.

Murine macrophages were detached using a cell scraper, and after centrifugation, a solution with a cell density of 70,000 cells mL^−1^ was prepared. An aliquot (300 μL) was then transferred into black microplates with a clear bottom (SPL Life Sciences (Pocheon-si, Republic of Korea)), and the microplates were incubated. Once the cells reached confluence, the medium was discarded, and the cells were rinsed with HBSS (2×, 100 μL). They were then incubated for 1 h with the extracts at the different concentrations (200 μL; 500–2000 μg mL^−1^). After the incubation period, the cells were washed with HBSS (2×, 100 μL), and a solution of 2,2′-azobis(2-methylpropionamide) dihydrochloride (AAPH) (100 μL; 600 μM) was added. Fluorescence readings were taken every 5 min for 1 h by using a FLX800 microplate reader (Agilent, Santa Clara, CA, USA) at 485 nm excitation and 538 nm emission. Quercetin was used as the positive control, while DCFH solution and DMEM were tested as negative controls. The results were expressed as the percentage of inhibition at the highest concentration tested (2000 μg mL^−1^) [99].

### 3.9. NO^•^ Production Inhibition Assay

The ability of samples to inhibit nitric oxide (NO^•^) production was assessed by using the method described by [100]. The essential oils and crude extracts were dissolved in H_2_O:DMSO (50:50, *v v*^−1^) and H_2_O, respectively, and successively diluted with water to obtain a range of concentrations for testing (8–0.125 mg mL^−1^). The murine macrophage cell line (RAW 264.7) was routinely maintained under the conditions described in Section 3.8. The cells were detached by using a cell scraper, and a solution with 5 × 10^5^ cells mL^−1^ was prepared and transferred to 96-well plates. After 24 h of incubation, the cells were exposed to the studied samples at different concentrations for 1 h. After this period, the cells were stimulated with lipopolysaccharide (LPS) (1 µg mL^−1^) (Sigma, St. Louis, MO, USA) for 24 h. Dexamethasone (50 µM) was used as the positive control, and cells with and without LPS served as negative controls.

Nitric oxide was measured using a Griess reagent kit (Promega Corporation, Madison, WI, USA). A standard curve for nitrite (sodium nitrite from 100 to 0.78 µM; y = 0.0068x + 0.0951; R^2^ = 0.9864) was prepared in a 96-well plate. After transferring 100 µL of the cell culture supernatant to a plate, the same volume of Griess reagent was added. The nitrite was quantified by measuring absorbance at 540 nm (ELX800 microplate reader, BioTek Instruments, Winooski, VT, USA) and comparing the absorbance with the standard calibration curve. The obtained results were presented as EC_50_ values, representing the sample concentration required to inhibit nitric oxide production by 50% in µg mL^−1^.

### 3.10. Antiproliferative Activity

The antiproliferative activity of the studied samples was assessed against four human tumor cells: gastric adenocarcinoma (AGS), colorectal adenocarcinoma (CaCo-2), breast adenocarcinoma (MCF-7), and lung carcinoma (NCI-H460). Additionally, the non-tumor cell line VERO (African green monkey kidney) was also tested. All cell lines used were acquired from the Leibniz Institute DSMZ—German Collection of Microorganisms and Cell Cultures GmbH (Braunschweig, Germany). The cell lines under investigation were regularly cultivated as adherent cultures in Gibco Roswell Park Memorial Institute (RPMI-1640) medium supplemented with the previously mentioned supplements (Section 3.8), except for the VERO cells, which were maintained in DMEM as described above. The samples studied were sequentially dissolved and diluted as described previously (Section 3.9). The concentrations tested ranged from 400 to 6.25 mg mL^−1^.

After trypsinization, cells were resuspended to a density of 1.0 × 10^4^ cells per well and transferred to 96-well plates, except for VERO cells, which were seeded at 1.9 × 10^4^ cells per well. The sulforhodamine B (Extra Synthesis, Genay, France) colorimetric assay was performed as previously described [101]. Ellipticine was used as the positive control, and the cells without samples as the negative control. The results were expressed as the sample concentration required to inhibit cell proliferation by 50% (GI_50_ values, µg mL^−1^).

The selectivity index (SI), the ratio of the cytotoxic concentration at 50% (GI_50_) for VERO cells to the tumor cells’ GI_50_, was also calculated using Equation (2).

SI = (GI_50_ of non-tumor cells)/(GI_50_ of tumor cells) (2)

### 3.11. In Vitro Determination of Sun Protection Factor (SPF)

The extracts were assessed for their in vitro sun protection factor (SPF), following a previously described methodology with modifications [102]. The extracts were redissolved in ethanol to prepare solutions at 0.050, 0.075, 0.100 and 0.125%. Absorbance measurements were performed in triplicate using a SpectraMax Plus 384 microplate reader (Molecular Devices, San Jose, CA, USA) over the wavelength range of 290 to 320 nm in 5 nm intervals. Ethanol served as the analytical blank. The SPF values were calculated according to Equation (3).

(3) $$SPF=CF\sum_{290}^{320}\;EE\left(\lambda \right)I\left(\lambda \right)Abs(\lambda )$$

where

*CF* = 10 (correction factor)
*EE*(*λ*) = erythemal effect spectrum
*I*(*λ*) = solar intensity spectrum
*Abs*(*λ*) = absorbance of the sample at wavelength λ

The *EE*(*λ*) × *I*(*λ*) values are constant [103].

#### Determination of the UVA/UVB Ratio and Critical Wavelength (λc)

The UVA/UVB ratio corresponds to the proportion of the area under the absorption curve in the UVA region (320–400 nm) relative to that in the UVB region (290–320 nm), as described by Wu et al. [66]. Ratios closer to 1.0 indicate greater UVA protection.

To determine this ratio, the extracts were redissolved in ethanol at concentrations of 0.050, 0.075, 0.100 and 0.125%. Absorbance spectra for each dilution were recorded in triplicate from 290 to 400 nm, at 5 nm intervals, using a SpectraMax Plus 384 microplate reader (Molecular Devices, San Jose, CA, USA). Ethanol was used as the analytical blank. The UVA/UVB ratios were computed based on Equation (4):

(4) $$UVA/UVB=\frac{\int_{320}^{400}A\left(\lambda \right)d\lambda /\int_{320}^{400}d\lambda }{\int_{290}^{320}A\left(\lambda \right)d\lambda /\int_{290}^{320}d\lambda }$$

where:

*A*(*λ*) = mean absorbance at each wavelength
*d*(*λ*) = wavelength interval between measurements

The photoprotective performance of the extracts in the UVA region was further classified according to Boot’s star rating system (Table 12) [65].

In addition, the absorption spectra were used to determine the critical wavelength (λc), defined as the wavelength below which 90% of the absorbance area is located. A critical wavelength equal to or greater than 370 nm is considered indicative of broad-spectrum UV protection [104].

### 3.12. In Silico Analyses of Metabolites Identified in the Crude Extract of B. dracunculifolia

The PubChem platform (https://pubchem.ncbi.nlm.nih.gov/, accessed on 18 July 2026) was used to obtain the canonical SMILES (Simplified Molecular Input Line Entry System) formulas for each secondary metabolite identified in the extract of the aerial parts of *B. dracunculifolia* (PubChem ID shown in Table 2). These canonical structures were used as input for the in silico analyses performed using the following open-access platforms: Way2Drug http://www.way2drug.com/passonline/predict.php (accessed on 10 September 2025), Swiss Target Prediction http://www.swisstargetprediction.ch/ (accessed on 24 April 2026), PANTHER Classification System http://www.pantherdb.org/ (accessed on 27 April 2026), SwissADME http://www.swissadme.ch/ (accessed on 12 September 2025), and ProTox 3.0 https://tox.charite.de/protox3/ (accessed on 16 September 2025).

#### 3.12.1. In Silico Study of PASS Prediction

The biological potential of secondary metabolites was predicted by comparing their structures with a comprehensive database of active molecules using the PASS Online program hosted on the Way2Drug computing platform [105]. This program assesses the biological activity of compounds, accounting for pharmacological characteristics, mechanisms of action, metabolic interactions, and enzyme transporters, among other factors. PASS Online is based on the structure-activity relationship and has an average forecasting accuracy of 96% [106]. This program presents a bioactivity score calibrated to Pa and Pi values, assigning an active prediction when a compound’s Pa value (possible activity) exceeds its Pi value (possible inactivity) [107]. The prediction of activity is based on the analysis of the structure-activity relationships of the training set, which contains more than 300,000 compounds exhibiting more than 4000 biological activities. The values of Pa and Pi range between 0.000 and 1.000. Only activities with Pa > Pi are considered possible for a specific compound. If Pa > 0.7, the substance is very likely to exhibit activity in experiments, but the probability of it being an analog of a known drug is also high. If 0.5 < Pa < 0.7, the substance is likely to exhibit activity in experiments, but the probability is lower, and the substance is different from known drugs. If Pa < 0.5, the substance is unlikely to exhibit activity in experiments. However, if this activity is confirmed in the experiment, the substance can be considered a new active chemical compound [96].

#### 3.12.2. Prediction of Compound-Related Target Genes and GO Enrichment Analysis

Potential molecular targets associated with the metabolites identified in the crude extract of *B. dracunculifolia* were predicted using the SwissTargetPrediction platform, considering *Homo sapiens* as the reference organism [68,69]. The predictions are based on structural similarity between the analyzed compounds and molecules with known biological targets. Only targets showing predicted interaction probabilities higher than 10 were selected for subsequent analyses.

Genes associated with oxidative stress/ROS, inflammation, and cancer were also retrieved from the GeneCards database https://www.genecards.org/ (accessed on 27 April 2026) [108]. These data were compared with the predicted targets obtained from SwissTargetPrediction to identify molecular targets potentially related to the biological activities evaluated experimentally in this study.

GO enrichment analysis was subsequently performed using the PANTHER Classification System http://www.pantherdb.org/ (accessed on 27 April 2026) [109], considering GO biological-process categories. Statistical overrepresentation was evaluated using Fisher’s exact test with false discovery rate (FDR) correction. Representative GO terms were selected according to statistical significance and biological relevance to the antioxidant, anti-inflammatory, antiproliferative, and photoprotective activities investigated in this study.

#### 3.12.3. In Silico Analysis of ADME Properties

The ADME properties of the identified phytoconstituents were evaluated using the online tool SwissADME [89]. This free tool assesses pharmacokinetics, drug similarity, and chemical compatibility of small molecules in medicinal chemistry. SwissADME relies on in silico models and utilizes various descriptors, including physicochemical parameters [molecular weight (g/mol), Csp^3^ fraction, number of rotatable bonds, hydrogen bond acceptors, hydrogen bond donors, molar refractivity (MR), topological polar surface area—TPSA (Å), CLogP/w (lipophilicity), and Log S (aqueous solubility)], pharmacokinetic parameters [gastrointestinal absorption, blood–brain barrier (BBB) permeation, P-gp substrate, Log *K_p_* (skin permeation, cm/s)], and drug-likeness parameters [Lipinski’s rule of five (RO5)].

This tool predicts bioavailability based on six physicochemical properties, such as lipophilicity, size, polarity, solubility, flexibility, and saturation, to detect drug similarity. The ADME properties of human passive gastrointestinal absorption (HIA) and blood–brain barrier (BBB) permeation, as well as substrate or non-substrate status for the permeability glycoprotein (P-gp), were determined as positive or negative using the BOILED-Egg model in the tool developed by Daina et al. [89]. The Lipinski filter (Pfizer) corresponds to the pioneering rule of five [109], and among other things, was incorporated into this tool for predicting drug similarity [89]. The bioavailability radar for predicting oral bioavailability was developed using the SwissADME tool and employing different physicochemical parameters [90].

#### 3.12.4. In Silico Prediction of Toxicity

The ProTox 3.0 web server [93] was used to predict multiple toxicity outcomes for the metabolites identified in this study, including hepatotoxicity, nephrotoxicity, neurotoxicity, cardiotoxicity, mutagenicity, carcinogenicity, immunotoxicity, and cytotoxicity. The expected LD_50_ (mg/kg) and toxicity classification were assessed for each phytochemical utilizing this platform.

### 3.13. Statistical Analyses

All analyses were performed in triplicate. The results were subjected to analysis of variance (ANOVA), and the differences between means were determined by Tukey’s test with a significance level of 5% using the Minitab 17 program.

## 4. Conclusions

This study provides a comprehensive phytochemical and biological characterization of the crude extract obtained from the aerial parts of *Baccharis dracunculifolia*. UHPLC-MS/MS analysis revealed a phenolic-rich profile dominated by phenylpropanoids and flavonoids, leading to the identification of 14 metabolites not previously reported in this species, significantly expanding its known chemical composition. Notably, the high abundance of phenylpropanoids, including coniferaldehyde, highlights this metabolite class as a major contributor to the chemical profile of the extract.

The biological investigations demonstrated that the extract exhibits pronounced cellular antioxidant and anti-inflammatory activities, reducing intracellular oxidation by 81% and inhibiting nitric oxide production in activated macrophages (IC_50_ = 62.00 µg/mL). These findings, together with its high flavonoid content and photoprotective activity observed in vitro, indicate its ability to modulate oxidative stress- and inflammation-related processes. In contrast, the antiproliferative activity was moderate, suggesting that the predominant biological effects of the extract are associated with cytoprotective rather than cytotoxic mechanisms.

The integration of quantitative phytochemical analysis, cell-based assays, and in silico target prediction enabled the establishment of coherent composition–activity relationships, supporting a multi-target mechanism associated with redox homeostasis, inflammatory regulation, apoptosis, and cellular responses to UV radiation. Overall, these findings expand the current knowledge of the chemical composition and biological properties of *B. dracunculifolia* and provide a scientific basis for future studies aimed at investigating its potential applications in pharmaceutical, nutraceutical, and photoprotective products. Further in vivo studies, mechanistic investigations, and formulation development will be necessary to confirm these prospects.

## Figures and Tables

**Table 1:** Quantitative analysis of secondary metabolites present in the crude extract of the aerial parts of *B. dracunculifolia* by UHPLC-MS/MS.

N	Compounds	Retention Time (min)	[M − H]^−^ (Precursor Ion > Fragment)	Concentration (µg/g)	CV	PubChem ID
Organic acids						
1	Malic acid	10.885	133.20 > 114.90	82.65 ± 1.07	1.3	525
2	Quinic acid	9.893	191.20 > 84.80	343.93 ± 3.34	0.97	37439
Coumarins						
3	Coumarin	9.444	146.80 > 90.95 *	<1	--	323
Phenolic aldehydes						
4	*p*-Hydroxybenzaldheyde	8.548	123.10 > 76.95 *	17.31 ± 0.33	1.9	126
5	Vanillin	8.711	151.40 > 136.05	13.40 ± 1.10	7.87	1183
6	Isovanillin	8.722	153.10 > 65.00 *	<1	--	12127
Phenolic acids						
7	Salicylic acid	9.781	137.40 > 92.90	<1	--	338
8	*p*-Hydroxybenzoic acid	8.246	137.20 > 92.90	266.46 ± 14.90	5.59	135
9	Protocatechuic acid	7.564	153.20 > 108.95	141.73 ± 1.87	1.32	72
10	α-Resorcylic acid	7.571	153.20 > 108.90	124.51 ± 6.71	5.39	7424
11	Vanillic acid	8.416	167.40 > 152.00	19.80 ± 1.24	6.29	8468
Phenylpropanoids						
12	Coniferaldehyde	9.086	178.90 > 90.95 *	>600	--	5280536
13	*p*-Coumaric acid	8.851	163.20 > 119.00	>600	--	637542
14	Ferulic acid	8.927	193.20 > 133.95	224.39 ± 7.16	3.19	445858
15	Caffeic acid	8.348	179.20 > 135.00	379.91 ± 0.59	0.16	689043
16	Chlorogenic acid	7.922	354.80 > 162.95 *	>600	--	1794427
Flavanones						
17	Naringenin	9.744	271.10 > 151.00	569.51 ± 5.25	0.92	439246
Flavones						
18	Chrysin	3.942	253.00 > 143.00	1.342 ± 0.004	0.27	5281607
19	Luteolin	3.557	286.90 > 152.85 *	<1	--	5280445
Flavanols						
20	(-)-Epicatechin	7.656	289.10 > 245.10	9.26 ± 0.84	9.04	72276
Flavonols						
21	Kaempferol	9.916	286.80 > 120.90 *	34.52 ± 0.17	0.49	5280863
22	Morin	9.719	302.80 > 152.90 *	>600	--	5281670
23	Quercetin	9.733	301.10 > 151.00	38.66 ± 2.46	6.36	5280343
24	Rutin	8.959	609.00 > 300.00	490.08 ± 19.44	3.97	5280805
Alkaloids and pyridinic acids						
25	Nicotinic acid	4.572	123.90 > 79.85 *	53.25 ± 2.05	3.85	938
26	Caffeine	8.602	194.90 > 137.95 *	6.69 ± 0.15	2.21	2519

* [M + H]^+^; CV: Coefficient of variation.

**Table 2:** Phenol and total flavonoid content of the crude extract of *B. dracunculifolia* aerial parts.

CE (mg/mL)	Total Phenol Content (µg Gallic Acid Equivalents/mg CE)	Flavonoid Content (µg Quercetin Equivalents/mg CE)
1.00	540.40 ± 6.37 ^a^	495.90 ± 9.35 ^ab^
0.75	350.36 ± 7.74 ^b^	472.00 ± 8.43 ^a^
0.50	195.46 ± 11.96 ^c^	507.79 ± 4.08 ^b^
0.25	83.62 ± 0.96 ^d^	515.70 ± 20.30 ^b^

Values are expressed as the mean ± standard deviation of two independent trials, each performed in triplicate, for the determination of total phenolic and total flavonoid contents. Data were subjected to analysis of variance (ANOVA), and differences among means were evaluated using Tukey’s test (*p* ≤ 0.05). Different letters within the same column indicate significant differences among means according to Tukey’s test (*p* ≤ 0.05). CE: Crude extract.

**Table 3:** Antioxidant activity determined by scavenging assays of the 2,2-diphenyl-1-picrylhydrazyl (DPPH^•^) radical and 2,2′-azino-bis-(3-ethylbenzothiazoline-6-sulfonic acid) (ABTS^•+^), and iron-reducing antioxidant power (FRAP) of the crude extract (CE) of the aerial parts of *Baccharis dracunculifolia*.

Sample	DPPH^•^ EC_50_ (mg mL/CE)	ABTS^•+^ (µM Trolox/mg CE)	FRAP (µM Ferrous Sulfate/mg CE)
CE	0.86 ± 0.07 ^b^	4.16 ± 0.12 ^b^	0.93 ± 0.04 ^b^
Quercetin	0.01 ± 0.01 ^a^	-	-
Trolox	-	0.25 ± 0.01 ^a^	9.17 ± 0.01 ^a^

Values are expressed as the mean ± standard deviation of three independent experiments, each performed in triplicate, for the determination of DPPH, FRAP, and ABTS antioxidant activities. Data were subjected to analysis of variance (ANOVA), and differences among means were evaluated using Tukey’s test (*p* ≤ 0.05). Different letters within the same column indicate significant differences among means according to Tukey’s test (*p* ≤ 0.05).

**Table 4:** Cellular antioxidant activity (CAA) and nitric oxide (NO^•^) production inhibition of the crude extract of *Baccharis dracunculifolia* aerial parts (CE) in murine macrophage cells.

Sample	Cellular Antioxidant Activity	
	Maximum Concentration Tested (µg/mL)	Inhibition at the Maximum Concentration Tested (%)
CE	2000	81%
Quercetin	2000	93%
	**Nitric Oxide Production Inhibition (IC_50_ µg/mL)**	
CE	62 ± 7 ^b^	
Dexamethasone	6.3 ± 0.4 ^a^	

Values are expressed as the mean ± standard deviation of the assay performed in triplicate for the determination of the 50% inhibitory concentration (IC_50_) of nitric oxide production. Data were subjected to analysis of variance (ANOVA), and differences among means were evaluated using Tukey’s test (*p* ≤ 0.05). Different letters within the same column indicate significant differences among means according to Tukey’s test (*p* ≤ 0.05).

**Table 5:** Antiproliferative activity of *Baccharis dracunculifolia* aerial part crude extract (CE).

Cell Lines	Antiproliferative Activity (GI_50_ µg/mL)		
	CE	SI	Ellipticine
AGS	165 ± 15 ^aB^	1.41	1.23 ± 0.03 ^A^
Caco-2	216 ± 5 ^bB^	1.08	1.21 ± 0.02 ^A^
MCF-7	255 ± 25 ^cB^	0.91	1.02 ± 0.02 ^A^
NCI-H460	257 ± 4 ^cB^	0.90	1.01 ± 0.01 ^A^
VERO	233 ± 11 ^abB^		1.41 ± 0.06 ^A^

The results represent the mean ± standard deviation of three independent trials performed in triplicate. Different lowercase letters in columns and uppercase letters in rows indicate a significant difference by Tukey’s test (*p* ≤ 0.05). GI_50_ = concentration that reduces proliferation by 50%; SI = selectivity index. Ellipticine was used as a positive control. AGS (gastric adenocarcinoma); Caco-2 (colorectal adenocarcinoma); MCF-7 (breast adenocarcinoma); NCI-H460 (lung carcinoma); VERO (African green monkey renal epithelial cells).

**Table 6:** Sun Protection Factor (SPF), UVA/UVB ratio, UVA protection, and critical wavelength (λc) of the crude extract of the aerial parts of *Baccharis dracunculifolia*.

CE (%)	SPF	UVA/UVB Ratio	Anti-UVA Protection	λc (nm)
0.050	7.79 ^d^ ± 0.27	1.31 ^a^ ± 0.01	Ultra	352
0.075	11.84 ^c^ ± 0.26	1.31 ^a^ ± 0.01	Ultra	350
0.100	15.84 ^b^ ± 0.16	1.30 ^a^ ± 0.01	Ultra	350
0.125	19.30 ^a^ ± 0.21	1.28 ^b^ ± 0.00	Ultra	350

The results are the mean ± standard deviation of three repetitions. Classification of UVA protection according to Boots’ star rating [65] (see Table in Section 3.11). Different letters within the same column indicate significant differences among means according to Tukey’s test (*p* ≤ 0.05). Critical wavelength ≥ 370 nm indicates broad-spectrum sun protection. CE: Crude extract of *B*. *dracunculifolia*.

**Table 7:** Predicted biological activities of identified compounds of *B. dracunculifolia* obtained by using the PASS online program.

Compounds	Type of Predicted Activity (Pa > Pi)							
	Antioxidant	Free Radical Scavenger	Lipid Peroxidase Inhibitor	Anti-Inflammatory	Apoptosis Agonist	Antineoplastic	Anticarcinogenic	Chemopreventive
Malic acid	--	--	0.370 > 0.044	0.567 > 0.004 ^a^	--	--	0.313 > 0.053	--
Quinic acid	0.830 > 0.003	0.541 > 0.008	0.627 > 0.008	0.705 > 0.015	0.328 > 0.111	0.699 > 0.026	0.587 > 0.013	0.489 > 0.015
Coumarin	0.389 > 0.013	0.472 > 0.012	0.393 > 0.037	0.615 > 0.028 0.382 > 0.028 ^a^	0.611 > 0.025	0.523 > 0.065 0.472 > 0.022 ^b^	0.324 > 0.050	0.364 > 0.027
*p*-Hydroxy- benzaldheyde	--	0.415 > 0.016	0.452 > 0.023	0.451 > 0.013 ^a^	0.653 > 0.020	0.549 > 0.057 0.375 > 0.036 ^b^	0.351 > 0.041	--
Vanillin	0.403 > 0.012	0.546 > 0.008	0.711 > 0.005	0.436 > 0.015 ^a^	0.705 > 0.014	0.636 > 0.038 0.466 > 0.023 ^b^ 0.310 > 0.038 ^d^	0.388 > 0.033	0.364 > 0.027
Isovanillin	0.403 > 0.012	0.546 > 0.008	0.711 > 0.005	0.436 > 0.015 ^a^	0.705 > 0.014	0.636 > 0.038 0.466 > 0.023 ^b^ 0.310 > 0.038 ^d^	0.388 > 0.033	0.364 > 0.027
Salicylic acid	0.318 > 0.020	0.504 > 0.010	0.420 > 0.030	0.713 > 0.014 0.812 > 0.002 ^a^	0.392 > 0.077	--	0.358 > 0.039	0.360 > 0.028
*p*-Hydroxy- benzoic acid	0.320 > 0.020	0.519 > 0.009	0.477 > 0.019	0.503 > 0.056 0.743 > 0.002 ^a^	0.387 > 0.079	--	0.370 > 0.036	0.356 > 0.028
Protocatechuic acid	0.401 > 0.012	0.420 > 0.030	0.516 > 0.015	0.707 > 0.003 ^a^ 0.538 > 0.046	0.462 > 0.049	--	0.387 > 0.033	0.374 > 0.025
α-Resorcylic acid	0.423 > 0.010	0.566 > 0.007	0.538 > 0.013	0.538 > 0.046 0.640 > 0.003 ^a^	0.484 > 0.043	--	0.412 > 0.029	0.397 > 0.023
Vanillic acid	0.374 > 0.014	0.643 > 0.005	0.564 > 0.011	0.505 > 0.055 0.720 > 0.002 ^a^	0.512 > 0.038	0.326 > 0.138	0.413 > 0.029	0.460 > 0.017
Coniferaldehyde	0.353 > 0.016	0.487 > 0.011	0.438 > 0.026	0.713 > 0.014 0.518 > 0.006 ^a^	0.871 > 0.005	0.551 > 0.056 0.322 > 0.020 ^b^	0.498 > 0.019	0.503 > 0.014
*p*-Coumaric acid	0.553 > 0.005	0.627 > 0.005	0.529 > 0.014	0.641 > 0.027 0.684 > 0.003 ^a^	0.661 > 0.019	0.520 > 0.065 0.394 > 0.033 ^b^	0.559 > 0.015	0.516 > 0.014
Ferulic acid	0.540 > 0.005	0.731 > 0.004	0.618 > 0.008	0.651 > 0.023 0.661 > 0.003 ^a^	0.702 > 0.015	0.601 > 0.045 0.467 > 0.023 ^b^ 0.303 > 0.028 ^c^ 0.318 > 0.020 ^d^	0.616 > 0.012	0.644 > 0.008
Caffeic acid	0.603 > 0.005	0.647 > 0.005	0.570 > 0.011	0.604 > 0.031 0.648 > 0.003 ^a^	0.711 > 0.014	0.530 > 0.063 0.399 > 0.032 ^b^	0.571 > 0.014	0.551 > 0.012
Chlorogenic acid	0.785 > 0.004	0.856 > 0.002	0.855 > 0.003	0.598 > 0.032 0.387 > 0.026 ^a^	0.589 > 0.028	0.778 > 0.014 0.391 > 0.033 ^b^ 0.358 > 0.030 ^d^	0.846 > 0.004	0.833 > 0.003
Naringenin	0.794 > 0.003	0.769 > 0.003	0.815 > 0.003	0.660 > 0.021	0.709 > 0.014	0.751 > 0.018 0.640 > 0.008 ^b^	0.724 > 0.008	0.724 > 0.006
Chrysin	0.708 > 0.004	0.693 > 0.004	0.677 > 0.005	0.637 > 0.025 0.344 > 0.043 ^a^	0.842 > 0.005	0.769 > 0.016 0.654 > 0.007 ^b^ 0.393 > 0.009 ^c^ 0.362 > 0.029 ^d^ 0.351 > 0.028 ^e^ 0.341 > 0.027 ^f^	0.618 > 0.012	0.576 > 0.011
Luteolin	0.775 > 0.004	0.755 > 0.003	0.740 > 0.004	0.661 > 0.021 0.343 > 0.044 ^a^	0.866 > 0.005	0.783 > 0.014 0.672 > 0.007 ^b^ 0.389 > 0.009 ^c^ 0.370 > 0.028 ^d^ 0.366 > 0.026 ^e^ 0.354 > 0.025 ^f^	0.690 > 0.009	0.648 > 0.008
(-)-Epicatechin	0.810 > 0.003	0.842 > 0.002	0.888 > 0.003	0.548 > 0.044	0.698 > 0.015	0.675 > 0.030 0.486 > 0.020 ^b^	0.795 > 0.005	0.788 > 0.004
Kaempferol	0.856 > 0.003	0.771 > 0.003	0.783 > 0.004	0.676 > 0.019	0.881 > 0.005	0.791 > 0.013 0.566 > 0.013 ^b^ 0.463 > 0.005 ^c^ 0.349 > 0.031 ^d^	0.715 > 0.008	0.669 > 0.008
Morin	0.850 > 0.003	0.759 > 0.003	0.783 > 0.004	0.680 > 0.018 0.312 > 0.062 ^a^	0.886 > 0.005	0.796 > 0.012 0.556 > 0.014 ^b^ 0.463 > 0.005 ^c^ 0.342 > 0.033 ^d^	0.709 > 0.008	0.677 > 0.007
Quercetin	0.872 > 0.003	0.811 > 0.003	0.788 > 0.004	0.689 > 0.017 0.303 > 0.068 ^a^	0.887 > 0.005	0.797 > 0.012 0.577 > 0.012 ^b^ 0.447 > 0.005 ^c^ 0.355 > 0.030 ^d^	0.757 > 0.007	0.717 > 0.006
Rutin	0.923 > 0.003	0.988 > 0.001	0.987 > 0.001	0.728 > 0.013	0.747 > 0.011	0.849 > 0.007 0.536 > 0.016 ^b^ 0.344 > 0.014 ^c^ 0.443 > 0.018 ^d^	0.983 > 0.001	0.968 > 0.001
Nicotinic acid	--	--	--	--	--	--	--	--
Caffeine	--	--	--	0.459 > 0.070 0.457 > 0.012 ^a^	--	--	--	--

Pa = Probable activity; Pi = Probable inactivity; ^a^ (intestinal); ^b^ (breast cancer); ^c^ (small cell lung cancer); ^d^ (lung cancer); ^e^ (colorectal cancer); ^f^ (colon cancer).

**Table 8:** Predicted targets for identified compounds of *B. dracunculifolia* obtained by using the SwissTargetPrediction platform.

Molecular Targets	*p*-Coumaric Acid	Caffeic Acid	Naringenin	Chrysin	Luteolin	Kaempferol	Morin	Quercetin	Rutin
Targets associated with ROS/redox processes (probability %)									
MAOA	nan	nan	nan	27.70	100.0	65.80	14.31	100.0	nan
MAOB	nan	nan	36.27	11.2	nan	nan	nan	nan	nan
NOX4	nan	nan	10.06	27.70	100.0	100.0	21.42	100.0	44.88
XDH	nan	nan	nan	100.0	100.0	100.0	21.42	100.0	13.32
Targets associated with inflammatory processes (probability %)									
CXCR1	nan	nan	nan	nan	27.90	40.26	14.31	100.0	nan
F2	nan	nan	11.20	nan	27.90	40.26	11.94	100.0	nan
MMP9	20.85	73.93	10.06	12.78	100.0	65.80	14.31	100.0	nan
PTGS2	nan	nan	nan	27.70	38.62	27.08	11.94	nan	9.49
SYK	nan	nan	10.06	27.70	100.0	65.80	14.31	68.03	nan
Targets associated with cancer-related processes (probability %)									
AKT1	nan	nan	nan	nan	27.90	40.26	nan	100.0	nan
ALK	nan	nan	nan	11.20	27.90	40.26	14.31	100.0	nan
AXL	nan	nan	nan	11.20	27.90	40.26	14.31	100.0	nan
CDK1	nan	nan	nan	nan	30.38	40.26	14.31	100.0	nan
CDK1/CCNB1 CCNB2/CCNB3 *	nan	nan	nan	nan	100.0	51.79	12.73	53.81	nan
CDK2	nan	nan	10.06	nan	nan	47.68	12.73	49.85	nan
CDK5R5/CDK5	nan	nan	10.06	100.0	100.0	51.79	12.73	53.81	nan
CDK6	nan	nan	nan	100.0	60.06	47.68	12.73	49.85	nan
EGFR	nan	7.18	nan	15.92	27.90	40.26	11.15	100.0	nan
FLT3	nan	nan	nan	27.70	100.0	100.0	21.42	100.0	nan
MET	nan	nan	10.06	11.99	27.90	40.26	nan	100.0	nan
NEK6	nan	nan	nan	nan	27.90	40.26	14.31	100.0	nan
PIM1	nan	nan	nan	16.71	27.90	40.26	14.31	100.0	nan
PLK1	nan	nan	nan	11.20	27.90	40.26	11.15	100.0	nan
PTK2	nan	nan	nan	nan	27.90	40.26	11.15	100.0	nan
SRC	nan	nan	10.06	11.20	27.90	40.26	11.15	100.0	nan

“not a number” (for short, “nan”) indicates no prediction by the respective tool. * Cyclin-dependent kinase 1/cyclin B (kinase and other cytosolic protein).

**Table 9:** Representative enriched Gene Ontology (GO) biological-process terms associated with predicted targets of compounds identified in the crude extract from the aerial parts of *Baccharis dracunculifolia*.

GO Term	GO ID	FDR	Fold Enrichment	Functional Category
Response to oxidative stress	GO:0006979	3.09 × 10^−8^	10.77	Antioxidant/redox-ROS response
Cellular response to oxidative stress	GO:0034599	1.44 × 10^−9^	7.88	Antioxidant/redox-ROS response
Regulation of reactive oxygen species metabolic process	GO:2000377	1.52 × 10^−10^	10.97	Antioxidant/redox-ROS response
Cellular response to hydrogen peroxide	GO:0070301	8.31 × 10^−6^	12.65	Antioxidant/redox-ROS response
Prostanoid metabolic process	GO:0006692	3.81 × 10^−16^	34.30	Anti-inflammatory/eicosanoids-NO
Prostaglandin biosynthetic process	GO:0001516	3.06 × 10^−8^	36.11	Anti-inflammatory/eicosanoids-NO
Inflammatory response	GO:0006954	1.01 × 10^−10^	4.83	Anti-inflammatory/eicosanoids-NO
Regulation of inflammatory response	GO:0050727	3.82 × 10^−10^	5.56	Anti-inflammatory/eicosanoids-NO
Regulation of apoptotic process	GO:0042981	1.06 × 10^−26^	4.72	Antiproliferative/apoptosis signaling
Positive regulation of apoptotic process	GO:0043065	3.23 × 10^−10^	5.13	Antiproliferative/apoptosis signaling
Cell cycle G2/M phase transition	GO:0044839	2.83 × 10^−12^	21.97	Antiproliferative/apoptosis signaling
Response to UV-A	GO:0070141	2.72 × 10^−7^	39.20	Photoprotection/UV-radiation response
Cellular response to UV-A	GO:0071492	9.67 × 10^−5^	35.64	Photoprotection/UV-radiation response
Response to UV	GO:0009411	1.68 × 10^−5^	7.09	Photoprotection/UV-radiation response
DNA damage response	GO:0006974	1.30 × 10^−4^	2.83	Photoprotection/UV-radiation response

Terms were selected based on statistical significance (false discovery rate, FDR) and manually curated according to their biological relevance to the experimental endpoints evaluated in this study. Functional categories were assigned to reflect antioxidant/redox, anti-inflammatory, antiproliferative, and photoprotective activities.

**Table 10:** In silico physicochemical properties, pharmacokinetic profile, and drug-likeness characteristics of identified compounds of *B. dracunculifolia.*

Compounds	Molecular Weight (g/mol)	Molar Refractivity	TPSA (Å^2^)	Log *P*_o/w_ (WLOGP)	Log *S* (ESOL)	Qualitative Water Solubility	GI Absorption	BBB Permeant	*p*-gp Substrate	Log *K*_p_ (Skin Permeation) (cm/s)	Lipinski Rule	Bioavailability Score
Malic acid	134.09	26.05	94.83	−1.09	0.32	HS	High	N	N	−8.01	Y; 0 viol.	0.56
Quinic acid	192.17	40.11	118.22	−2.32	0.53	HS	Low	N	N	−9.15	Y; 0 viol.	0.56
Coumarin	146.14	42.48	30.21	1.79	−2.29	S	High	Y	N	−6.20	Y; 0 viol.	0.55
*p*-Hydroxy- benzaldheyde	122.12	33.85	37.30	1.20	−1.87	VS	High	Y	N	−6.09	Y; 0 viol.	0.55
Vanillin	152.15	40.34	46.53	1.21	−1.82	VS	High	Y	N	−6.37	Y; 0 viol.	0.55
Isovanillin	152.15	40.34	46.53	1.21	−1.67	VS	High	Y	N	−6.54	Y; 0 viol.	0.55
Salicylic acid	138.12	35.42	57.53	1.09	−2.50	S	High	Y	N	−5.54	Y; 0 viol.	0.85
*p*-Hydroxy- benzoic acid	138.12	35.42	57.53	1.09	−2.07	S	High	Y	N	−6.02	Y; 0 viol.	0.85
Protocatechuic acid	154.12	37.45	77.76	0.80	−1.86	VS	High	N	N	−6.42	Y; 0 viol.	0.56
α-Resorcylic acid	154.12	37.45	77.76	0.80	−1.67	VS	High	N	N	−6.63	Y; 0 viol.	0.56
Vanillic acid	168.15	41.92	66.76	1.10	−2.02	S	High	N	N	−6.31	Y; 0 viol.	0.85
Coniferaldehyde	178.18	50.06	46.53	1.50	−2.04	S	High	Y	N	−6.31	Y; 0 viol.	0.55
*p*-Coumaric acid	164.16	45.13	57.53	1.38	−2.02	S	High	Y	N	−6.26	Y; 0 viol.	0.85
Ferulic acid	194.18	51.63	66.76	1.39	−2.11	S	High	Y	N	−6.41	Y; 0 viol.	0.85
Caffeic acid	180.16	47.16	77.76	1.09	−1.89	VS	High	N	N	−6.58	Y; 0 viol.	0.56
Chlorogenic acid	354.31	83.50	164.75	−0.75	−1.62	VS	Low	N	N	−8.76	Y; 1 viol.: OH > 5	0.11
Naringenin	272.25	71.57	86.99	2.19	−3.49	S	High	N	Y	−6.17	Y; 0 viol.	0.55
Chrysin	254.24	71.97	70.67	2.87	−4.19	MS	High	Y	N	−5.35	Y; 0 viol.	0.55
Luteolin	286.24	76.01	111.13	2.28	−3.71	S	High	N	N	−6.25	Y; 0 viol.	0.55
(-)-Epicatechin	290.27	74.33	110.38	1.22	−2.22	S	High	N	Y	−7.82	Y; 0 viol.	0.55
Kaempferol	286.24	76.01	111.13	2.28	−3.31	S	High	N	N	−6.70	Y; 0 viol.	0.55
Morin	302.24	78.03	131.36	1.99	−3.16	S	High	N	N	−7.05	Y; 0 viol.	0.55
Quercetin	302.24	78.03	131.36	1.99	−3.16	S	High	N	N	−7.05	Y; 0 viol.	0.55
Rutin	610.52	141.38	269.43	−1.69	−3.30	S	Low	N	Y	−10.26	N; 3 viol.: MW > 500, O > 10, OH > 5	0.17
Nicotinic acid	123.11	31.20	50.19	0.78	−1.26	VS	High	Y	N	−6.80	Y; 0 viol.	0.85
Caffeine	194.19	52.04	61.82	−1.03	−1.48	VS	High	N	N	−7.53	Y; 0 viol.	0.55

MR: Molar Refractivity; TPSA: topological polar surface area. Log S: aqueous solubility (Scale: <−10 < Poorly < −6 < Moderately < −4 < Soluble < −2 Very < 01; Log *K_p_*: Negative Log *k_p_* values suggest limited skin penetration, with a greater negative value indicating reduced permeation potential. Log *P*: A negative value for log *P* means the compound has a higher affinity for the aqueous phase (it is more hydrophilic); When log *P* = 0, the compound is equally partitioned between the lipid and aqueous phases; a positive value for log *P* denotes a higher concentration in the lipid phase (i.e., the compound is more lipophilic). HS = Highly soluble; VS = Very Soluble; S = Soluble; MS = Moderately Soluble. Lipinski rule: MW ≤ 500, MLOGP ≤ 5, N or O ≤ 10, NH or OH ≤ 5. Y: yes. N: no. viol.: violation.

**Table 11:** Oral, organ toxicity, and toxicological endpoint predicted activities of identified compounds of *B. dracunculifolia* obtained by using the ProTox-III web server.

Compounds	Oral Toxicity Prediction		Organ Toxicity (% Probability)					Toxicity Endpoint (% Probability)			
	Predicted LD_50_ (mg/kg)	Predicted Toxicity Class	Hepato-Toxicity	Neuro-Toxicity	Nephro-Toxicity	Cardio-Toxicity	Respiratory Toxicity	Carcino-Genicity	Immuno-Toxicity	Muta-Genicity	Cyto-Toxicity
Malic acid	2497	V	– (90)	– (98)	+ (55)	– (86)	– (64)	– (71)	– (99)	– (97)	– (74)
Quinic acid	9800	VI	– (80)	– (93)	+ (52)	– (53)	– (50)	– (73)	– (99)	– (94)	– (78)
Coumarin	196	III	– (69)	+ (58)	+ (56)	– (74)	– (93)	+ (83)	– (99)	– (53)	+ (56)
*p*-Hydroxy- benzaldheyde	2250	V	– (55)	– (57)	– (54)	– (77)	– (90)	+ (50)	– (99)	– (99)	– (83)
Vanillin	1000	IV	– (52)	– (59)	+ (53)	– (79)	– (98)	– (60)	– (55)	– (98)	– (94)
Isovanillin	1510	IV	– (52)	– (59)	+ (53)	– (79)	– (98)	– (60)	– (55)	– (98)	– (94)
Salicylic acid	1034	IV	+ (51)	– (69)	+ (78)	– (71)	+ (58)	– (67)	– (99)	– (98)	– (86)
*p*-Hydroxy- benzoic acid	2200	V	– (52)	– (76)	+ (68)	– (96)	– (61)	– (51)	– (99)	– (99)	– (86)
Protocatechuic acid	2000	IV	– (59)	– (86)	+ (61)	– (90)	– (58)	+ (72)	– (99)	– (97)	– (90)
α-Resorcylic acid	2000	IV	– (58)	– (86)	+ (72)	– (61)	– (52)	– (70)	– (99)	– (99)	– (88)
Vanillic acid	2000	IV	– (55)	– (77)	+ (64)	– (76)	– (77)	– (64)	– (97)	– (96)	– (93)
Coniferaldehyde	1560	IV	+ (50)	– (55)	+ (51)	– (85)	– (97)	– (63)	+ (79)	– (85)	– (90)
*p*-Coumaric acid	2850	V	– (51)	– (71)	+ (66)	– (98)	– (62)	+ (50)	– (91)	– (93)	– (81)
Ferulic acid	1772	IV	– (51)	– (74)	+ (62)	– (85)	– (77)	– (61)	+ (91)	– (96)	– (88)
Caffeic acid	2980	IV	– (57)	– (83)	+ (59)	– (97)	– (59)	+ (78)	– (50)	– (98)	– (86)
Chlorogenic acid	5000	V	– (72)	– (89)	+ (56)	– (99)	+ (57)	– (68)	+ (99)	– (93)	– (80)
Naringenin	2000	IV	– (67)	– (84)	+ (61)	– (79)	+ (82)	– (62)	– (88)	– (83)	+ (59)
Chrysin	3919	V	– (68)	– (86)	+ (60)	– (63)	+ (75)	– (62)	– (99)	– (57)	– (87)
Luteolin	3919	V	– (69)	– (89)	+ (62)	– (99)	+ (83)	+ (68)	– (97)	+ (51)	– (99)
(-)-Epicatechin	10,000	VI	– (72)	– (90)	+ (62)	– (99)	+ (79)	– (51)	– (96)	– (55)	– (84)
Kaempferol	3919	V	– (68)	– (89)	+ (62)	– (91)	+ (83)	– (72)	– (96)	– (52)	– (98)
Morin	3919	V	– (68)	– (89)	+ (62)	– (91)	+ (83)	– (72)	+ (52)	– (52)	– (98)
Quercetin	159	III	– (69)	– (89)	+ (62)	– (99)	+ (83)	+ (68)	– (87)	+ (51)	– (99)
Rutin	5000	V	– (80)	– (89)	+ (77)	– (98)	+ (63)	– (91)	+ (98)	– (88)	– (64)
Nicotinic acid	3720	V	+ (80)	+ (57)	+ (54)	– (67)	+ (73)	– (95)	– (99)	– (99)	– (77)
Caffeine	127	III	– (97)	+ (96)	– (73)	– (98)	+ (62)	– (93)	– (98)	– (94)	– (83)

Class I: fatal if swallowed (LD_50_ ≤ 5); Class II: fatal if swallowed (5 < LD_50_ ≤ 50); Class III: toxic if swallowed (50 < LD_50_ ≤ 300); Class IV: harmful if swallowed (300 < LD_50_ ≤ 2000); Class V: may be harmful if swallowed (2000 < LD_50_ ≤ 5000); Class VI: non-toxic (LD_50_ > 5000); (+) means active or toxic; (–) means inactive or nontoxic.

**Table 12:** Degrees of anti-UVA protection according to the Boots star rating system.

UVA/UVB Limits						
	0–0.2	0.21–0.4	0.41–0.6	0.61–0.8	0.81–0.9	>0.91
**Number of stars**	-	*	**	***	****	*****
**UVA protection**	Very low	Moderate	Good	Superior	Maximum	Ultra

## Data Availability

Data is contained within the article.
